# Relationship between Composition and Environmental Degradation of Poly(isosorbide-*co*-diol oxalate) (PISOX) Copolyesters

**DOI:** 10.1021/acs.est.2c09699

**Published:** 2024-01-26

**Authors:** Yue Wang, Kevin van der Maas, Daniel H. Weinland, Dio Trijnes, Robert-Jan van Putten, Albert Tietema, John R. Parsons, Eva de Rijke, Gert-Jan M. Gruter

**Affiliations:** † van‘t Hoff Institute for Molecular Sciences (HIMS), University of Amsterdam, Science Park 904, Amsterdam 1098 XH, The Netherlands; ‡ Institute for Biodiversity and Ecosystem Dynamics (IBED), University of Amsterdam, Science Park 904, Amsterdam 1098 XH, The Netherlands; § Avantium Support BV, Zekeringstraat 29, Amsterdam 1014 BV, The Netherlands

**Keywords:** biodegradable plastic, biobased, renewable, marine-degradable polyester, hydrolysis, isosorbide, oxalic acid, structure property relation

## Abstract

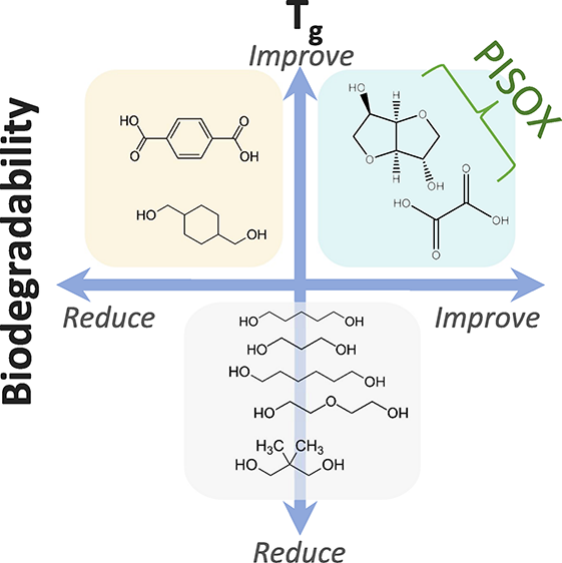

To reduce the global CO_2_ footprint of plastics, bio- and CO_2_-based feedstock are considered the most important design features for plastics. Oxalic acid from CO_2_ and isosorbide from biomass are interesting rigid building blocks for high *T*_g_ polyesters. The biodegradability of a family of novel fully renewable (bio- and CO_2_-based) poly(isosorbide-*co*-diol) oxalate (PISOX-diol) copolyesters was studied. We systematically investigated the effects of the composition on biodegradation at ambient temperature in soil for PISOX (co)polyesters. Results show that the lag phase of PISOX (co)polyester biodegradation varies from 0 to 7 weeks. All (co)polyesters undergo over 80% mineralization within 180 days (faster than the cellulose reference) except one composition with the cyclic codiol 1,4-cyclohexanedimethanol (CHDM). Their relatively fast degradability is independent of the type of noncyclic codiol and results from facile nonenzymatic hydrolysis of oxalate ester bonds (especially oxalate isosorbide bonds), which mostly hydrolyzed completely within 180 days. On the other hand, partially replacing oxalate with terephthalate units enhances the polymer’s resistance to hydrolysis and its biodegradability in soil. Our study demonstrates the potential for tuning PISOX copolyester structures to design biodegradable plastics with improved thermal, mechanical, and barrier properties.

## Abstract 2

This work shows the potential for tuning the composition of biodegradable PISOX copolyesters, for optimizing the resulting properties (thermal-, mechanical-, barrier-, hydrolysis-, and biodegradability) to target certain applications, such as polymer coating of controlled-release fertilizers, films, and rigids for packaging, 3D printing, etc.

## Introduction

Currently, the majority of plastics used are derived from fossil resources, and their production typically consumes fossil energy. For future plastics, more sustainable feedstock will be required, e.g., biomass or CO_2_ (via carbon capture and utilization (CCU)) or from recycled waste materials. By changing feedstock, an important fraction of the 1 Gt 2022 global plastic-related CO_2_ emissions can be reduced, and when using CO_2_ as feedstock, even negative emissions for plastics are feasible.^1^ Furthermore, biomass and CO_2_ can provide a very wide range of building blocks (monomers) for polymer synthesis, and potentially plastics with comparable or even better properties (compared to traditional fossil-based plastics) can be produced.^2^

Since (bio)degradability will be a very important design parameter for many future plastic materials, the environmental biodegradability of novel polymers should be considered in earlier phases of their development. Although recycling is preferred within the context of a circular economy, it is not always feasible. Many regions lack the infrastructure for proper waste management, and even highly advanced systems cannot prevent all plastic leakage into the environment, such as in fisheries and polymer coating of controlled-release fertilizers.^3,4^ The fact that nonrecyclable single-use packaging has no value after use leads to a high chance of these materials ending up in the environment. Because of the resistance of conventional plastics to (bio)degradation, they will eventually fragment into micro- and nanosized particles (MNPs). These can interact with (and bind) pollutants present in the environment, which increases the MNPs concern as this could promote migration and accumulation of pollutants in the food chain worldwide.^5^ Therefore, the next generation of plastics should at least be either closed-loop recyclable when collection infrastructure is available or designed to degrade completely (e.g., mineralize) over time when (unavoidably) ending up in the environment. (Bio)degradable plastics should not be considered as a solution to the littering problem but rather to avoid the endless buildup of plastics in the environment.

Isosorbide is a bicyclic secondary diol derived from sugar. When incorporating it in polymers, its structure provides rigidity and therefore favorable thermomechanical properties to these polymers. Isosorbide’s asymmetric structure reduces the resulting polymer’s crystallinity, making isosorbide a good candidate for producing (biobased) amorphous polyesters (or polycarbonates) with a high glass transition temperature (*T*_g_),^6,7^ for instance as a replacement for poly(ethylene terephthalate) (PET). Oxalic acid can be potentially obtained from CO_2_.^8,9^ Polyoxalates, i.e., polymers derived from oxalic acid or its esters, show susceptibility to (nonenzymatic) hydrolysis and subsequently could undergo fast biodegradation in various environments (with moisture present).^10^ Therefore, a family of novel renewable poly(isosorbide-*co*-diol oxalate) (PISOX-diol) polyesters was developed by our group, aiming for materials with high *T*_g_ in combination with biodegradability.^11,12^

These PISOX copolyesters have good mechanical, water vapor- and oxygen-barrier and thermal properties. Specifically, their tensile properties are better than, or at least comparable to, those of high-performance polymers such as Eastman’s TRITAN or acrylonitrile butadiene styrene (ABS).^12^ Also, oxygen barrier properties are comparable to those of polyethylene 2,5-furandicarboxylate (PEF) and thus considerably better than those of PET (∼10×), which could be critical for (food) packaging.^12−14^ Additionally, the *T*_g_ of the PISOX copolyesters is tunable from subzero to 167 °C (for the PISOX homopolymer) by varying the type and amount of codiol.^11,12^

The biodegradability in soil and marine environments at ambient temperature (25 °C) of a representative PISOX, poly(isosorbide-*co*-1,6-hexanediol) oxalate with a 75/25 IS/HDO molar ratio (PISOX-HDO25), was reported previously.^10^ It was found that PISOX-HDO25 mineralized faster than cellulose in both environments (after 50 days). This high-level biodegradability was related to the relatively fast nonenzymatic hydrolysis of polyoxalates. Analogously, we expected PISOX copolymers with other diols to also biodegrade rapidly in soil under ambient conditions. They should then also be home compostable, as in that case the conditions are expected to be more favorable for degradation.^15^ Overall, the combination of good thermomechanical properties (e.g., *T*_g_ > 100 °C) and fast biodegradability (also in marine environments) is unique and could make this family of copolyesters ideal for short-term applications that demand amorphous polymers with very good mechanical- and thermal properties.^12^ Moreover, studying the relationship between compositions and degradation of copolymers allows us to tailor the structure of PISOX copolyesters for the optimal combination of properties for various applications.

The aim of this research is to assess the biodegradability and hydrolyzability of this series of novel PISOX-diol (co)polyesters at ambient temperature (25 °C) in soil and water and investigate the relationship between structure/composition and biodegradation/hydrolysis. Specifically the effects of (1) the molar ratio of isosorbide to codiol, (2) the codiol type and chain length and (3) replacing part of the oxalate with an aromatic building block (terephthalate) in addition to the diol (poly(isosorbide-*co*-1,3-propylene oxalate-*co*-terephthalate; PISOXT-diol) were studied.

## Materials and Methods

### Materials

**Figure 1:**
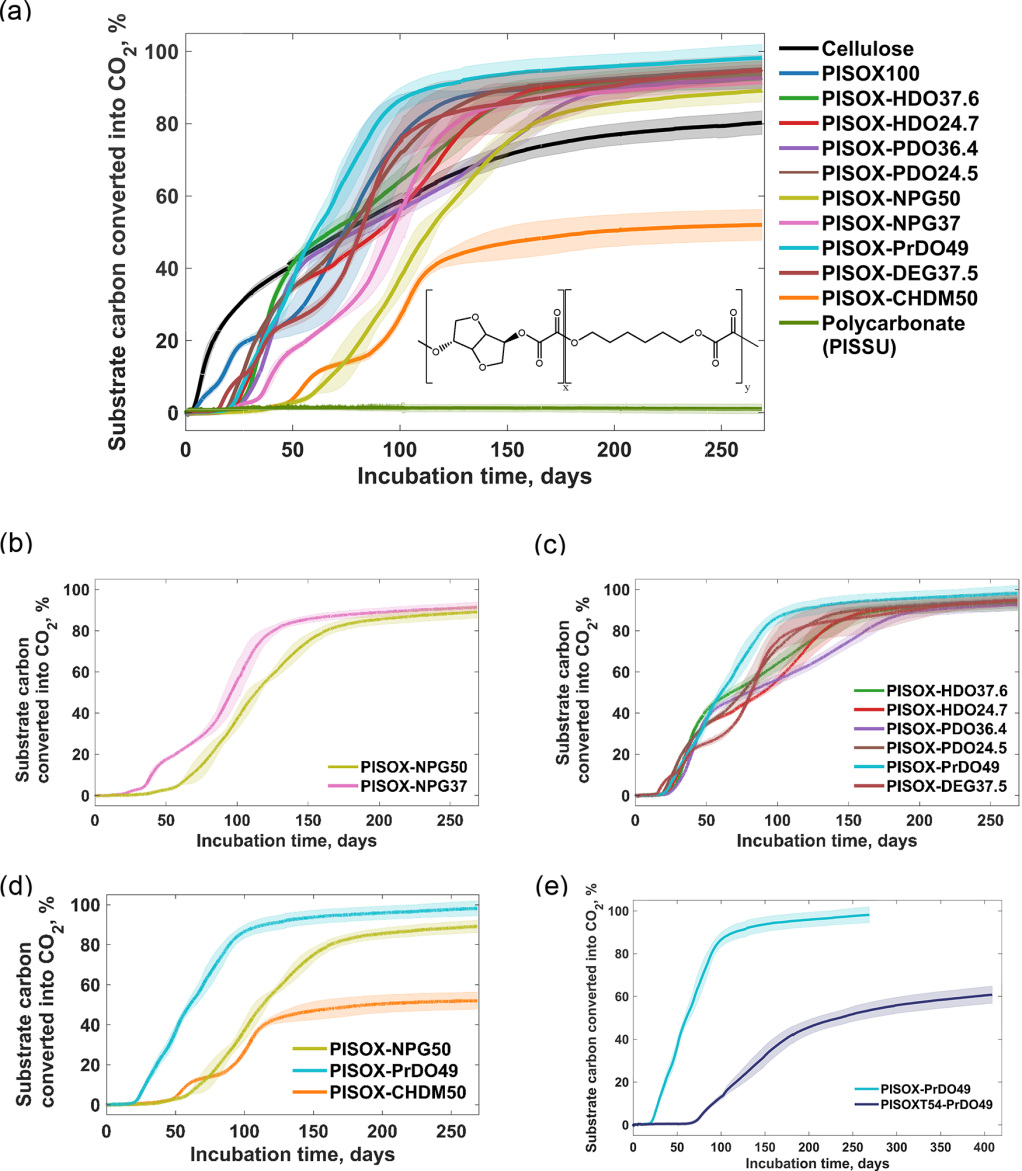
Biodegradation curves of PISOX, PISOX copolyesters, PISOXT54-PrDO49, cellulose, and polycarbonate (positive and negative reference) with approximately 5 mg (substrate) carbon per gram of dry soil at 25 °C. Mean biodegradation (lines) were plotted. The shaded area represents the standard deviation of at least three replicates for each polymer composition. (a) Chemical structure of the poly(isosorbide-*co*-1,6-hexanediol) oxalate (PISOX-HDO) is shown as an example (inset). PISSU behaved similarly to the negative reference (no biodegradation).^16^ (b) Comparison of degradation profiles for PISOX-NPG with the same codiol (neopentyl glycol) but different isosorbide content. Data for other types of codiol can be found in Figure S6. (c) Comparison of degradation profiles for copolyesters containing linear codiols. (d) Comparison of degradation profiles for copolymers with similar codiol ratios ratio (50/50). 25/75 and 37.5/62.5 are represented in Figure S8. (e) Comparison of degradation profiles for PISOX-PrDO49 and PISOXT54-PrDO49.

Cellulose (powder, 20 μm average particle size) and polycarbonate (PC) were purchased from Sigma-Aldrich. PISOX, PISOX-diols, PISOXT-diol, and poly(isosorbide succinate) (PISSU) were synthesized by our research group,^12,16^ and their compositions are listed in Table 1. The chemical structure of the PISOX-HDO copolymer is depicted in Figure 1 as an example. The synthesis and characterization of a 1,4-cyclohexanedimethanol (CHDM) oxalate oligomer model compound is provided in the Supporting Information.

**Table 1:**
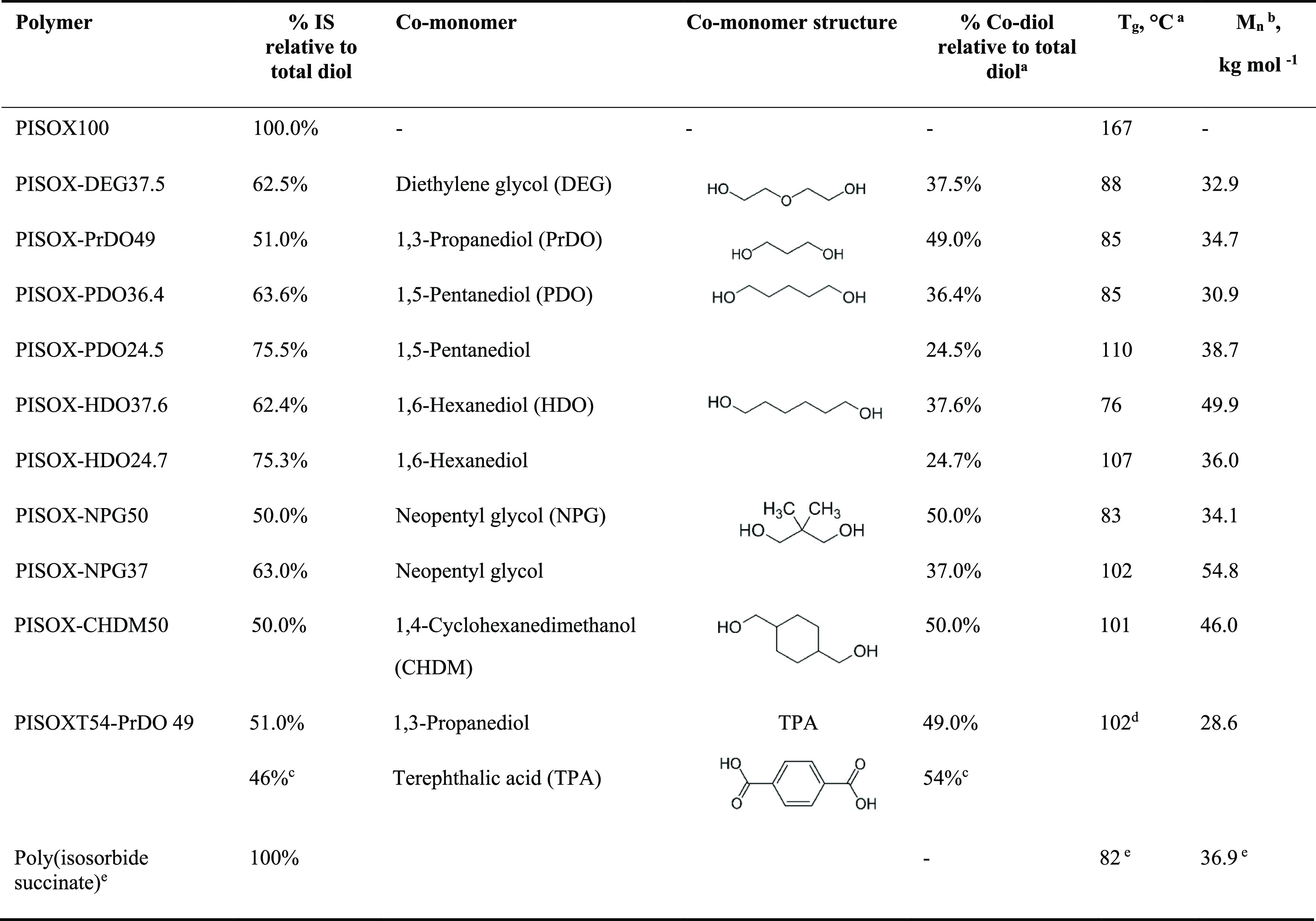
Overview of the Composition of Copolymers Evaluated in This Study^12,16^

a Values taken from ref (12).

b Number-average molecular weights (*M*_n_), method described in SI following Figure S12.

c % Oxalic acid (46%) or TPA (54%) relative to total diacid.

d Measured value with the same method described in ref (12).

e Values taken from ref (16).

Deuterated water (99.9% D) and dimethyl sulfoxide (DMSO, ≥99.9%) were purchased from Aldrich and Fisher Scientific, respectively, and used for the hydrolysis experiments.

### Soil

The standard soil (LUFA 2.2) used for the biodegradation experiments was obtained from LUFA Speyer and was stored at room temperature before the experiments. The properties of the soil are given in Table S1. The moisture level of the soil was adjusted to about 50% of the water-holding capacity (WHC) by slowly adding a mineral salt solution (Table S2) to the soil in soft plastic bags. These plastic bags were massaged and preconditioned at 25 °C for about 1 week. The pH of the soil was measured after moisture adjustment.

### Biodegradation Testing Method

A respirometer (Respicond) was employed to perform biodegradation tests. The details of the method have been described in a previous publication.^17^ In short, biodegradation tests were conducted in a system with 95 parallel sealed 250 mL vessels maintained at 25 °C in the dark. The CO_2_ released from the soil was captured by a potassium hydroxide solution (KOH) placed inside each of the vessels. The conductivity of the KOH solution, which decreased with trapping more CO_2_, was measured hourly and was used to determine the amount of evolved CO_2_. Subsequently, the percentage conversion of substrate carbon to CO_2_ was calculated by subtracting the amount of CO_2_ evolved from the blanks (soil only) and dividing the resulting amount by the theoretical maximum amount of CO_2_ that could be released from each polymer sample after complete conversion to CO_2_.

Two series of experiments were performed, and the incubations lasted approximately 270 and 410 days, respectively. Each series includes two abiotic controls, six replicates of blanks (soil without test material), triplicates of cellulose (positive references), and three to five replicates of each of the PISOX copolyesters. Triplicates of polycarbonate were used as negative references only in the 270 days experiments. Most polymers were tested in the 270 days series. PISOXT54-PrDO49 was tested in the 410 days series.

All polymers were ground and sieved through a 600 μm mesh filter, except cellulose powder, which was used as obtained. Typically, 120–170 mg of test material (equivalent to approximately 75 mg of carbon) was added on top of 19 g of wet soil (equivalent to 15 g of dry soil) in each vessel. The resulting carbon loading was kept at 5 mg C g^–1^ dry soil in all experiments with added polymers.

**Figure 2:**
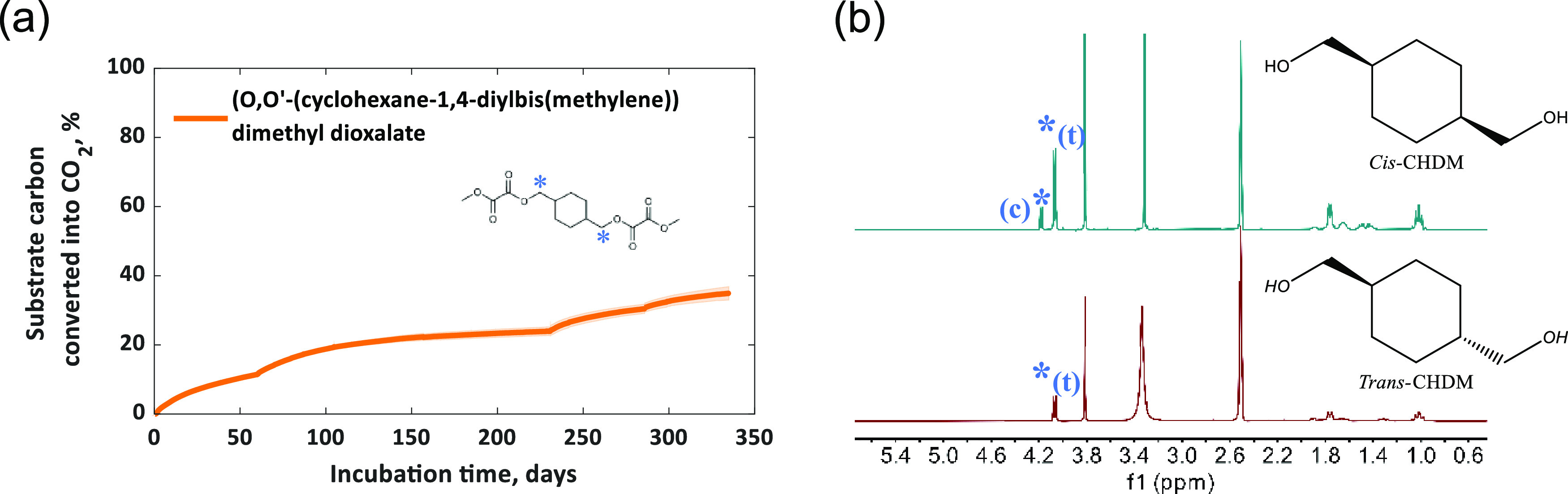
(a) Biodegradation curve of the oligomer model compound (*O*,*O*′-(cyclohexane-1,4-diylbis(methylene)) dimethyl dioxalate) at 25 °C in soil. Mean biodegradation (line) was plotted. The shaded area represents the standard deviation of four replicates. (b) ^1^H NMR spectra of the model compound before (upper) and after (lower) biodegradation in DMSO-d6. The (*c*) and (*t*) symbols represent assigned peaks for the *cis* and *trans* isomers of the corresponding protons (*) in the chemical structure.

Additionally, CHDM and the oligomer model compound *O*,*O*′-(cyclohexane-1,4-diylbis(methylene)) dimethyl dioxalate (MeOX-CHDM-OXMe; see Figure 2) were tested in a following experiment (335 days) under the same conditions. CHDM was tested in a lower concentration, which corresponded to the molar proportion in the evaluated polymers (50% relative to all diol). This resulted in a final carbon loading of approximately 2.2 and 5.3 mg C g^–1^ dry soil for CHDM and the model compound, respectively.

### Hydrolysis

About 10 mg of polymer powder (<600 μm) was added to a nuclear magnetic resonance (NMR) tube, followed by 1 mL D_2_O solution with 3.6 mM DMSO as the internal standard. The tubes were sealed and kept in an incubator at 25 °C. A Bruker Avance III 400 MHz NMR spectrometer was used to measure (^1^H NMR) soluble hydrolysis products over time over a period of 185 days (except for the PISOX-HDO37.6 for 112 days). The soluble monomers were further quantified to determine the degree of hydrolysis of the polyesters. The calculations are described in our previous study.^17^ All hydrolysis experiments were performed in triplicate.

## Results and Discussion

### Overview of PISOX Biodegradability in Soil

The biodegradation of PISOX and its copolyesters with 10 different compositions at 25 °C in soil was followed over time, together with cellulose and polycarbonate as positive and negative references, respectively (Figure 1a). The biodegradation curves of PISOX and its copolyesters with noncyclic codiol clearly show a high level of degradation (more than 86%) after 270 days, which was higher than for cellulose (80 ± 3%). The PISOX-CHDM50, which consists of 25% molar ratio of 1,4-cyclohexanedimethanol (50% codiol), is the only exception, showing less than 50% degradation after 270 days. As expected, no biodegradation of polycarbonate (a negative reference) was observed.

The shapes of the curves show the different phases of the biodegradation. The lag phase reflects initial hydrolysis and possible microbial adaptation to the released hydrolyzed oligomers and monomers. The subsequent increase in CO_2_ release indicates the mineralization of the oligomers and monomers. The lag phase varies from 3 to 43 days (Figure S17), while the time to biodegradation completion (curves reaching plateau, daily mineralization rate <0.1 mg) for (co)polyesters ranges between 120 and 240 days.

These results demonstrate the competitive biodegradability of PISOX compared with other biodegradable polyesters. For example, poly(lactic acid) (PLA) is industrially compostable; however, it biodegrades very slowly at ambient temperature.^17−19^ The mineralization of polybutylene succinate (PBS) particles (average size of 158 μm) was reported to be over 80% after 74 days under industrial composting conditions,^20^ while limited mineralization of PBS (dumbbell-shaped) was observed in a 6 month incubation at 25 °C (leveled off at 65% after 100 days).^21^ Despite the different shapes of the specimens in these two studies, (bio)degradation of PBS was found to be relatively slow at ambient temperature.^22,15,23,24^ Boyandin et al. observed that buried polyhydroxyalkanoate (PHA) films (0.1 mm thick), commercially available polyhydroxybutyrate (PHB) and polyhydroxybutyrate-*co*-hydroxyvalerate (PHBV), lost 14–98% of their total mass within 250 days under natural tropical conditions (∼29 °C, 75% relative humidity).^25^ With comparable and even better biodegradability than these examples, PISOX copolyesters with a high *T*_g_ (76–110 °C), very good mechanical and gas barrier properties can extend the possible applications for biodegradable plastics, particularly to replace fossil-based counterparts such as polystyrene (PS) and PET.^26,27^

### Effect of Oxalic Acid and Isosorbide

Biodegradation curves of PISOX copolyesters with the same type of codiol (i.e., PDO, HDO, and NPG) but different IS content are compared (Figure S6). In the case of PISOX with PDO and NPG, the compositions with higher isosorbide content exhibit faster degradation. However, in the case of PISOX with HDO, there is essentially no difference in the degradation behavior between the two compositions tested. This discrepancy appears to be caused by a combination of the type and content of the comonomer.

Besides the effect of the IS incorporated, molecular weights are also commonly considered to affect biodegradation. It is noteworthy to mention that the molecular weight of PISOX-NPG37 is much higher (∼1.5× *M*_n_; ∼2× *M*_w_) than that of PISOX-NPG50 (Table 1). However, PISOX-NPG37 shows both a shorter lag phase and faster biodegradation (Figure 1b). Apparently, IS-OX ester bonds (with secondary alcohol) hydrolyze faster than NPG-OX ester bonds (with primary alcohol). The hydrolysis results (Figures S14 and S15) also support this conclusion. Furthermore, there seems to be no obvious trend of biodegradation rate as a function of the molecular weights and polydispersity index (PDI) of the copolyesters studied (Figure S7).

Returning to the discussion of the possible effect of IS on biodegradation, Qi et al. suggested that steric hindrance caused by the butterfly bicyclic structure of isosorbide could impede hydrolysis of polyesters containing isosorbide, while conversely the strong hydrophilicity/hygroscopicity of isosorbide could facilitate hydrolysis.^6^ The results shown in Figure S6 would suggest that the type of codiol could determine which factor is predominant for a certain PISOX type; however, no hard conclusions can be drawn on the effect of these competing factors because of the limited amount of compositions tested in this study. Although the final effect of isosorbide content (∼12.5% difference) on biodegradation was limited for most PISOX compositions, the homopolyester clearly showed the shortest lag phase (3 days) (Figure 1). This suggests that a higher isosorbide content favors faster biodegradation, likely due to increased water availability within the polymer matrix.

However, oxalic acid plays a more significant role in the biodegradability of the polyesters than isosorbide. It is supported by the fact that high molecular weight poly(isosorbide succinate) (PISSU), which has isosorbide as the only diol, did not exhibit measurable biodegradation in the same experiments.^16^ This can be attributed to the lower p*K*_a_ (higher acidity) of oxalic acid (p*K*_a_ 1.24 versus 4.2 for succinic acid) and the structure of oxalate esters, where two ester functional groups are directly adjacent. The proximity of carbonyl groups increases each other’s electrophilicity, thereby accelerating their hydrolysis.^26^ In contrast, this observation is not in line with those of Qi et al., who observed that the order of (enzymatic and nonenzymatic) hydrolysis rate was poly(isosorbide succinate) (100% IS) > copolyester (IS/1,4-butanediol 20/80)> PBS (0% IS), and higher content of isosorbide had more impact on PISSU with the presence of enzyme.^6^ The most obvious explanations for this are that the *M*_n_ of PISSU used by Qi et al. was much lower than that used in our studies (7.3 vs 36.9 kg mol^–1^) and that the applied temperature was higher than in our study (37 vs 25 °C). Given the fact that isosorbide is asymmetric, it reduces the crystallinity of the polyesters and when the content is high enough it will lead to amorphous polymers, which is beneficial for their biodegradability due to the increased accessibility of the ester bonds.^6,17^

It is worth mentioning that Qi et al. also found that the increase in isosorbide almost linearly correlates to *T*_g_,^6^ which is in line with the observation in our previous studies.^11,12^

### Effect of the Codiol Structure

To investigate the effect of the codiol type on the biodegradability of PISOX, copolyesters with different codiols in similar ratios (i.e., 25/75, 37.5/62.5, 50/50) were compared (Figure S8). We found that the mineralization of all PISOX containing linear codiols was similar (*p* > 0.05) (Figures 1c and S9e). This indicates that variations in intermediate chain length of the codiol (i.e., C3 to C6, ratio from 25 to 50%) have little effect on the biodegradability of PISOX copolyesters in terms of complete mineralization.

At the same time, a relatively short lag phase was observed for PISOX-DEG37.5 (Figures 1c and S17). Even though the difference is small, this suggests that the incorporation of (more) DEG is likely to make the polymers more biodegradable (i.e., (enzymatically) hydrolyzable). Similarly, Haernvall et al. observed that 2,5-furandicarboxylic acid (FDCA) and 5-sulfoisophthalic acid–based copolymers that contained ether diols were more susceptible to enzymatic hydrolysis than those containing alkyl diols.^28^ They suggested that the presence of oxygen (in ether diols) affected the interaction between the polymer and enzyme.

**Figure 3:**
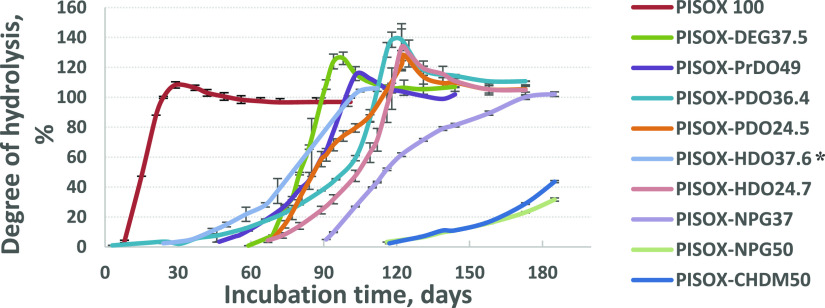
Degree of hydrolysis for PISOX and PISOX copolyesters in D_2_O versus time at 25 °C. The points represent the averages of triplicate experiments, with the error bars representing the standard deviation. * Hydrolysis of PISOX-HDO37.6 was run for 112 days instead of 180 days.

In contrast to the above, the final mineralization was significantly different for the group of PISOX-diol 50% (Figures 1d and S9c). The NPG codiol (at 50% content) shows slower biodegradation (Figure 1a) and hydrolysis (Figure 3). This may be attributed to the higher steric hindrance (relative to the linear structure) as a result of the two methyl branches. Furthermore, the negative effect of diol substituents with side chains on the (basic) hydrolysis was also reported for PET copolyesters.^29^

CHDM is often used for the development/modification of polymers to improve thermal stability and impact strength.^30,31^ For this reason, a PISOX composition with 50% CHDM was also made. When comparing its biodegradation to PISOX-NPG50, the lag phase and biodegradation behavior until around 40% conversion to CO_2_ are similar (Figure 1d). However, past this point, the biodegradation of PISOX-CHDM50 stalled and leveled off at almost 50% conversion to CO_2_, while the NPG variant continued to biodegrade. This is also in line with the observation that white residue particles remained on top of the soil for the PISOX-CHDM50 samples (Figure S10). In the PISOX-CHDM50, oxalic acid and isosorbide account for 56% of the carbon content, which means that at full biodegradation of this fraction, a conversion to CO_2_ of around 50% is expected. This indicates that the relatively low mineralization for PISOX-CHDM50 resulted from the presence of the cyclic codiol, which possibly does not convert to CO_2_. It is supported by the ^1^H NMR spectrum of the remaining particles that peaks related to isosorbide (5.5–4.5 ppm) disappeared, and only peaks related to CHDM were observed (Figure S11).

Comparison of the molecular weight distribution of PISOX-CHDM50 before and after biodegradation shows a significant decrease in molecular weights (from 46.0 to 1.0 kg mol ^–1^) (Figure S12). The two peaks (*M*_n_, 1312 and 743 g mol ^–1^) of the residues could be assigned to oligomers containing 7 and 4 units of CHDM (e.g., CHDM-OX-CHDM-OX-CHDM-OX-CHDM, 741 g mol ^–1^).

Even though CHDM was registered as readily biodegradable (OECD 301),^32^ we did not observe biodegradation of CHDM (2.2 mg C g^–1^ dry soil) in soil after 335 days, which is in line with the limited mineralization of PISOX-CHDM50. In biodegradation experiments of the oligomer model compound (*O*,*O*′-(cyclohexane-1,4-diylbis(methylene)) dimethyl dioxalate) CO_2_ evolution started immediately but only reached approximately 35% of the theoretical CO_2_ release (Figure 2) in 335 days. The nonbiodegradable CHDM represents 57% of the carbon of this model compound, and the results suggest that the model compound did not hydrolyze completely. In addition, the bumps on the biodegradation curves occurred after adding water (to compensate for moisture loss via evaporation/condensation), e.g., around 60, 230, and 290 days), which also indicates that the availability of water in soil limits the hydrolysis rate and subsequently the biodegradation of the model compound. Adding water directly may not be the ideal method to maintain soil moisture levels, but evaporation/condensation cannot be avoided in the closed vessels used in these experiments. Adding water to the soil is a common practice in standard biodegradation experiments as outlined in ASTM D-5988 and ISO-17556, which recommend adding a suitable amount of water to the test soil to bring its water content back to its initial value. The bumps on the biodegradation curves of the model compound (Figure 2a) were not observed for PISOX (co)polyesters, possibly due to isosorbide, which could enhance the hydrophilicity of PISOX polyesters, facilitating more effective water absorption from the soil (and facilitating subsequent hydrolysis) compared to CHDM based polyesters.

It is interesting to note that the *cis*/*trans* ratio of the model compound is 21/79 (Figure S1); however, only the *trans* ester was observed after biodegradation (Figure 2b). This suggests that the *cis* isomer is more susceptible to biodegradation (hydrolysis) than its *trans* isomer. It is in line with the low *cis*/*trans* ratio of the remaining PISOX-CHDM50 (Figure S11). The biodegradability of novel polymers containing CHDM should be of concern in terms of their fate in nature and release of nonbiodegradable micro/nanoplastics. Further investigation should therefore be conducted to assess the biodegradability of CHDM-containing polyesters in various environments, including soil.

### Effect of Aromatic Monomers

In order to explore the potential application of PISOX copolyesters with reduced hydrolysis rate (allowing longer shelf life), half of the oxalate in PISOX-PrDO49 was replaced with terephthalic acid (TPA) to obtain PISOXT54-PrDO49. Generally, the introduction of rigid aromatic monomers, like terephthalate, has been reported to result in an increased resistance to biodegradation.^33,2^ Although the terephthalate building block is not biobased, it could be obtained from PET recycling, i.e., from carbon above the ground. In addition, this replacement could (in the short term when new monomers are still relatively expensive) also reduce the cost of the feedstock for PISOX copolyesters.

The biodegradability of PISOXT54-PrDO49 was tested in soil at 25 °C. After more than 400 days of incubation, 61 ± 4% of PISOXT54-PrDO49 was converted into CO_2_ (Figure 1e). As was expected, compared to PISOX-PrDO49, the lag phase of the biodegradation curve for PISOXT54-PrDO49 was notably prolonged (over three times to 70 days). This resulted from replacing easily hydrolyzable ester bonds (i.e., oxalic esters) with aromatic acid esters (i.e., TPA).^10,33^ Esters of stronger acids form faster but also hydrolyze faster.

At the point biodegradation curves of PISOXT54-PrDO49 leveled off (daily mineralization rate <0.1 mg) after approximately 250 days, white residue particles were observed on top of the soil until the end of the incubation experiments after 410 days (Figure S10). The molecular weight (*M*_n_) of PISOXT54-PrDO49 decreased significantly (to 1.4 kg mol ^–1^) after biodegradation. In contrast to the curve of CHDM containing PISOX in Figure 1d, the curve of PISOXT54-PrDO49 in Figure 1e is still going up, which means that biodegradation is still progressing. It is therefore likely to continue increasing in time, but it is not certain that it will biodegrade completely.

The mineralization of TPA monomer in soil was reported to be around 40% after 7 weeks at 20 °C, which was comparable to linear aliphatic monomers, including 1,6-hexanediol, succinic acid, and glucose, tested in the same study.^34^

However, relatively large oligomers with TPA could be resistant to biodegradation at room temperature in soil. Witt et al. observed that the short oligomers, i.e., P–T–P (P: 1,3-propanediol, T: TPA) and P–T–P–T–P, were degraded within 8 weeks, whereas larger oligomers in the mixture were not degraded.^35^ They also studied the degradation of the oligomer mixtures under industrial composting (4 and 12 weeks) and aqueous conditions (5 weeks), where the size-exclusion chromatography profiles of oligomer mixtures demonstrated that a fraction of the larger oligomers degraded. Therefore, these results suggest that the relatively low temperature (lower than industrial composting) and the limited availability of water (compared to aqueous) in the soil hinder the hydrolysis of the larger oligomers. Subsequently, they cannot be taken up and mineralized by microorganisms.

Similarly, the incomplete mineralization of PISOXT54-PrDO49 could be explained by the nonbiodegradable oligomers containing TPA. PISOXT54-PrDO49 is a random copolyester, which means that T–P–T–P–T or oligomers with more TPA units could be hydrolysis products. The ^1^H NMR spectra of residues suggest that almost all oxalate ester bonds were hydrolyzed, and the residues mainly consisted of TPA, IS, and PrDO (Figure S13). This is different for PISOX-CHDM50 residues, for which isosorbide as a building block could not be observed.

Another aliphatic-aromatic copolyester containing TPA, poly(butylene adipate-*co*-terephthalate) (PBAT), is commercially available and is well-known for its biodegradability. Complete biodegradation of PBAT was observed in industrial compost.^35^ However, it is found that the biodegradation rate reduced significantly and is rather dependent on soil type at room temperature.^36^ For example, Han et al. studied the mineralization of PBAT film buried in four types of soil at 30 °C, and 0.3–16% of PBAT was converted to CO_2_ after 120 days.^36^ The highest mineralization percentage is comparable to that of PISOXT54-PrDO49, which was approximately 20% after 120 days.

To summarize, the incorporation of TPA could impede the hydrolysis of PISOX copolyesters due to the steric hindrance of the cyclic structure. Although oxalate esters could still undergo hydrolysis in soil later (than other PISOX (co)polyesters), the relatively large oligomers containing several TPA units (e.g., T–P–T–P–T) are difficult to hydrolyze further. As a result, they cannot be taken up by microorganisms and instead remain as residues in the soil.

### Nonenzymatic Hydrolysis

The results of the hydrolysis of PISOX (co)polyesters in pure water (D_2_O) are shown in Figure 3. The PISOX homopolymer shows by far the fastest hydrolysis with complete hydrolysis within 60 days. Most of the PISOX copolymers were hydrolyzed completely within 160 days, with the exception of the copolymers with NPG and CHDM, and the PISOXT54-PrDO49 (data not shown). In the case of PISOX-CHDM50 hydrolysis was only observed via ^1^H NMR spectra (i.e., formation of hydrolysis products) but not visually, which is different for PISOX-NPG50 where the decrease in the amount of particles was visually observed.

From the graph, it is clear that most of the curves at some point remarkably exceed 100% yield, which is not supposed to happen. The experiments were performed with DMSO as the internal standard, and this phenomenon therefore indicates a lower-than-designed amount of the standard present in the solution (detectable phase), leading to an overestimation of the product/standard ratio. Interestingly the values dropped back to around 100% with time, which indicates that the standard peak recovered. A plausible explanation is therefore that interaction between the internal standard DMSO and the insoluble polymers and/or oligomers takes place and decreases the DMSO concentration in solution. Subsequently, the concentration of soluble diols was overestimated and resulted in an overshoot. After the insoluble polymers and/or oligomers were hydrolyzed completely, all DMSO was released back into the solution and eventually normalized the degree of hydrolysis to approximate 100%. Although this interaction could result in less accuracy of the quantification of soluble monomers in especially the earlier stages of hydrolysis, it is still clear that polyesters hydrolyzed completely within the time frame of the experiments. In addition, the hydrolysis curve of PISOX-HDO 24.7 is consistent with PISOX HDO 25/75 from our previous study where the peak and the regression to ∼100% were observed around 120 and 150–160 days, respectively.^10^

In general, this trend of hydrolysis rates for the different PISOX diol compositions is consistent with the biodegradation trend. This indicates that (nonenzymatic) hydrolysis can play an important role in the biodegradation of amorphous polyesters in general and of PISOX (co)polyesters specifically.

No soluble hydrolysis products of PISOXT54-PrDO49 were observed in the ^1^H NMR spectra after 180 days of incubation at 25 °C, which suggests PISOXT54-PrDO49 is much more resistant to nonenzymatic hydrolysis than the other PISOX (co)polyesters. Meanwhile, over 40% of the polymer was converted to CO_2_ when incubated with soil. This much slower nonenzymatic hydrolysis rate indicates that enzymatic hydrolysis was dominant in biodegradation of PISOXT54-PrDO49 in soil, especially, considering the relatively reduced availability of water in soil versus the experiments in water.

In addition, the individual yields in the time of hydrolysis products (i.e., isosorbide and codiol for PISOX copolyesters) provide more insight into the hydrolysis mechanisms. For instance, the higher yield of diol indicates a higher susceptibility of the diol-oxalate bonds for hydrolysis. Specifically, isosorbide oxalate (IS-OX) ester bonds across the polymer were hydrolyzed and isosorbide released at a higher rate than the NPG-OX (Figure S14a) and CHDM-OX ester bonds (Figure S15). On the other hand, the relative yields of DEG are higher than for IS in the growth phase, which suggests that DEG-OX ester bonds are more susceptible to hydrolysis than IS-OX ester bonds (Figure S14b). The longer lag phase of PISOX-DEG37.5 than of PISOX100 could be explained by less hydrophilic copolyesters resulting from DEG (Figure 3). Additionally, the fact that the isosorbide and codiol, including PrDO, PDO, and HDO curves show essentially the same trend suggests that they are distributed randomly within the polymer structure (Figures S14c and S15).

## Applications and Implications

The biodegradability of PISOX copolyesters with noncyclic codiols, including 1,3-propanediol, diethylene glycol, 1,5-pentanediol, 1,6-hexanediol, and neopentyl glycol, are similar. The incorporation of more diethylene glycol could facilitate and more neopentyl glycol could impede biodegradation/hydrolysis of copolyesters, respectively (Figure S17). Moreover, no obvious trend was observed for the intermediate chain length of the diol (C3–C6) on biodegradability of PISOX copolyesters or for the molecular weight of the PISOX (co)polyesters.

The incorporation of cyclic building blocks is a typical strategy to improve the thermal and physical properties of polyesters. However, in the case of these being too apolar (hydrophobic), this may hinder the biodegradation/hydrolysis of the copolyesters, such as the PISOX copolyesters containing 50% 1,4-cyclohexanedimethanol and PISOXT54-PrDO49 containing 50% terephthalate. These materials could thus have an increased risk, with respect to microplastic accumulation and retention in the environment.

Isosorbide (derived from glucose) also has a cyclic structure and can thus provide good thermal (i.e., *T*_g_) and mechanical properties to polymers while also showing a high level of (bio)degradability.^37^ Further studies with more variation of isosorbide content (e.g., from 10 to 90%) could be interesting,^6^ although lower isosorbide content will result in lower *T*_g_ of the polymer if it is replaced with less rigid diols.^12^

In addition, oxalic acid, as a potentially future CO_2_-based and currently commercially available building block, shows potential regarding the design of biodegradable polyesters with enhanced mechanical and thermal (i.e., *T*_g_) properties and a possible negative carbon footprint.

The nonenzymatic hydrolysis results demonstrate the potential of PISOX (co)polyesters (excluding compositions with CHDM and TPA) to degrade completely in the aquatic environment within a reasonable time frame. They hydrolyze into monomers, which are expected to (readily) biodegrade.^10^ This is highly relevant for their potential marine degradability, and they should be interesting materials for home-composting disposal (e.g., when recycling is difficult with laminated paper or food-contaminated packaging). Additionally, for hydrolyzable PISOX (co)polyesters, their MNPs could theoretically undergo more facile hydrolysis compared to macroplastics (there is relatively more surface area in MNPs). This process of breakdown into oligomers and monomers will persist in the presence of moisture. As a result, the application of PISOX (co)polyesters may lead to a more limited presence of MNPs in the environment compared to nonhydrolyzable polymers, thereby contributing to the mitigation of negative effects associated with MNPs.

Generally, the outstanding biodegradability of PISOX copolyesters, in both soil and aquatic environments with sufficient moisture, makes them promising for short-term applications demanding good mechanical, thermal, and gas barrier properties.

Examples of potential applications include polymer coatings for example for controlled-release fertilizers, fishing gear, (food) packaging film, containers, and more. However, further investigation is required for the validation of these applications, with each requiring different polymer properties.

The tunability of PISOX copolyesters’ biodegradation behavior and solubility (for solution coating processes) could make the PISOX polymers interesting for paper coating or biodegradable coatings for controlled-release fertilizers.^38^ Specifically, when compared to PISOX homopolyesters, incorporating more NPG could slow down the decomposition rate of the coating, subsequently delaying the release of the fertilizer. The release behavior of coated fertilizers in soil is crucial and requires further investigation.^38,39^

PISOX (co)polyesters, especially those with DEG, offer good thermal and mechanical properties,^12^ making them suitable for fishing gear applications. In particular, their higher tensile strength (derived from isosorbide), compared to traditional biodegradable nets such as the blends of PBS and PBAT, provides advantageous performance.^12,40^

When fishing gear enters the aquatic environment, the tunable nonenzymatic hydrolysis of PISOX (co)polyesters will reduce ghost fishing gear and thus minimize this deadliest form of marine plastic as well as minimizing endless MNP accumulation in marine environments. As a downside, of course, the biodegradability of fishing gear may also shorten the application time window. There the chemical recyclability of polyesters such as PISOX may help make fishing gear more circular.

Low oxygen permeability (OP, indicating better barrier properties) is essential for certain food packaging applications.^14,41,42^ A lower OP results in a slower ingress of oxygen inside packaged food products, leading to oxidation and respiration, which is particularly relevant for fruits and vegetables.^40^ Therefore, PISOX (co)polyesters, with outstanding barrier properties and transparency (they are amorphous),^12^ can be utilized particularly for food packaging in geographies where plastic collection and recycling are challenging (either due to lack of accessible infrastructure or when single-use is desired for sanitation). In that case, home compostable packaging can be a desired end-of-life option.

Even though nonenzymatic hydrolysis may result in a limited shelf life for these materials (“plastics with an expiry date”), this is a trade-off between convenience and environmental risk reduction. Besides, fast nonenzymatic hydrolysis can also be used for efficient chemical recycling of these PISOX (co)polyesters via hydrolysis.

## Supporting Information Available

NMR spectra results; properties of soil; constituents of mineral salts solution used to adjust soil moisture; biodegradation curves for PISOX copolyesters; molecular weights; boxplots of the biodegradation percentages for PISOX copolyesters; photos of polymers before and after incubation; GPC chromatograms; individual yield of monomers, isosorbide, and codiol; percentages of dissolved NPG and DEG relative to the total amount of hydrolyzed diols in time; and overview of the lag phase for PISOX-based (co)polyesters biodegradation curves (PDF)
