# Supplementary material for: Relationship between Composition and Environmental Degradation of Poly(isosorbide-co-diol oxalate) (PISOX) Copolyesters
Source: Environ Sci Technol. 2024 Jan 26;58(5):2293–302. doi: 10.1021/acs.est.2c09699 (PMC10851428; doi:10.1021/acs.est.2c09699)
Supplement: Supplementary file 1 — es2c09699_si_001.pdf [file es2c09699_si_001.pdf]

Supporting Information

The relationship between composition and environmental degradation of poly(isosorbide oxalate) (PISOX) copolyesters

Yue Wang,[†, §] *Kevin van der Maas,*[†] *Daniel H. Weinland,*[†] *Dio Trijnes,*[†] *Robert-Jan van Putten,*[‡] *Albert Tietema,*[§] *John R. Parsons,*[§] *Eva de Rijke,*[§] *and Gert-Jan M. Gruter*[†,‡,*]

[†]van 't Hoff Institute for Molecular Sciences (HIMS), University of Amsterdam, Science Park 904, 1098 XH Amsterdam, the Netherlands

[§]Institute for Biodiversity and Ecosystem Dynamics (IBED), University of Amsterdam, Science Park 904, 1098 XH Amsterdam, the Netherlands

[‡]Avantium Support BV, Zekeringstraat 29, 1014 BV Amsterdam, the Netherlands;

Author e-mail addresses: y.wang6@uva.nl; k.vandermaas@uva.nl; d.h.weinland@uva.nl; dio.trynes@gmail.com; j.r.parsons@uva.nl; a.tietema@uva.nl; e.derijke@uva.nl; robert-jan.vanputten@avantium.com

* Correspondence: g.j.m.gruter@uva.nl

Summary: 22 pages, 17 figures, 2 tables.

Table of Content

Synthesis and characterisation of CHDM model compound (O,O'-(cyclohexane-1,4-diylbis(methylene)) dimethyl) S1

Figure S. 1 ^{1}H NMR spectrum of O,O'-(cyclohexane-1,4-diylbis(methylene)) dimethyl dioxalate in DMSO-d6...S3

Figure S. 2 ^{13}C NMR spectrum of O,O'-(cyclohexane-1,4-diylbis(methylene)) dimethyl dioxalate in DMSO-d6...S4

Figure S. 3 ^{1}H-^{1}H COSY NMR spectrum of O,O'-(cyclohexane-1,4-diylbis(methylene)) dimethyl dioxalate in DMSO-d6. ....S5

Figure S. 4 ^{1}H-^{13}C HSQC NMR spectrum of O,O'-(cyclohexane-1,4-diylbis(methylene)) dimethyl dioxalate in DMSO-d6. ....S6

Figure S. 5 ^{1}H-^{13}C HMBC NMR spectrum of O,O'-(cyclohexane-1,4-diylbis(methylene)) dimethyl dioxalate in DMSO-d6. ....S7

Table S 1. Properties of soil. S8

Table S 2. Constituents of mineral salts solution used to adjust soil moisture (OECD, 2014). S8

Figure S. 6 Biodegradation curves for PISOX copolyesters with 1,6-hexanediol (HDO) (a), 1,5-pentanediol (PDO) (b) and neopentyl glycol (NPG) (c) as co-diol. Mean biodegradation (lines) were plotted and the shaded area represents the standard deviation of at least four replicates for each polymer composition. S9

Figure S. 7 The molecular weights (M_n,(a); M_w, (b)) v.s. the time to plateau of mineralisation (daily mineralisation rate < 0.1 mg) (average of replicates) for PISOX copolyesters. Error bars represent 20% difference respectively. PISOX is not included in the figure as it is insoluble in DCM; it plateaus at 130 days. (c) The polydispersity index (PDI) v.s. the lag phase of mineralisation (average of replicates) for PISOX copolyesters. Corresponding Mn and Mw values (in kg mol^{-1}) are labeled in square brackets [] and parentheses () respectively. S11

Figure S. 8 Biodegradation curves of PISOX copolyesters with the similar co-diol ratio (25/75 (a), 37.5/62.5 (b), and 50/50 (c)) at 25 °C in soil. Mean biodegradation (lines) were plotted. The shaded area represents the standard deviation of at least three replicates. S12

Figure S. 9 Boxplots of the biodegradation percentages for PISOX copolyesters with 25% (a), 37.5% (b), 50% (c), all 25% & all 37.5% & PrDO 49% (d), all linear (e) co-

diol, respectively. Highlighted group in (d) was excluded in (e). The central red mark, the bottom and top edges of the box indicate the median, and the 25th and 75th percentiles, respectively. The lines extending from the box represent the variability outside the upper and lower quartiles. Corresponding ANOVA table shown under the graphs. S13

Figure S. 10 Photos of polymers before and after incubation. Visible particles left for PISOX-CHDM50 (a) and PISOXT54-PrDO49 (b) after incubation. Disappearance of particles and growth of microorganisms was observed for PSIXO-PDO36.4 (c). S15

Figure S. 11 ^{1}H NMR spectra of 1,4-cyclohexanedimethanol (in DCM-d2), PISOX-CHDM50 (in TCE, from[1]) and the residual of PISOX-CHDM50 after biodegradation (in DCM-d2). The *cis*/*trans* ratios of CHDM-monomer (3.44-3.56) and CHDM-esters (4.10-4.25 ppm) are 21/79 and 9/91 respectively. S15

Figure S. 12 GPC chromatograms of PISOX-CHDM50 and PISOXT54-PrDO49 before and after biodegradation. S16

Figure S. 13 ^{1}H NMR spectrum of PISOXT54-PrDO49 after biodegradation in DCM-d2 ......S17

Figure S. 14 Individual yield of monomers, isosorbide and co-diol for PISOX-DEG37.5 (a), PISOX-NPG37 (b) and PISOX-PDO36.4 (c) during 6-month hydrolysis at 25 °C in D_2O as percentage of their theoretical maximum release. Error bars represent standard deviation of triplicate hydrolysis experiments. * Percentages of dissolved NPG and DEG relative to the total amount of hydrolysed diols in time are shown in Figure S. 16. ......S18

Figure S. 15 Individual yield of monomers, isosorbide and co-diol for PISOX copolyesters during 6-month hydrolysis at 25 °C in D_2O as percentage of their theoretical maximum release. Error bars represent standard deviation of triplicate hydrolysis experiments. S20

Figure S. 16 Percentages of dissolved NPG and DEG relative to the total amount of hydrolysed diols in time. Triplicates were plotted. Dotted lines show the composition in the polymer. S21

Figure S. 17 Overview of the lag phase for PISOX-based (co)polyesters biodegradation curves. Error bars represent variation of replicates. S22

Synthesis and characterisation of CHDM model compound (O,O'-(cyclohexane-1,4-diylbis(methylene)) dimethyl)

Materials

1,4-cyclohexanedimethanol (99%) and triethylamine (99%) were purchased from Aldrich. Methyl oxalyl chloride was purchased from Thermo Fisher (97%). Tetrahydrofuran was purchased from VWR. DMSO-d6 was purchased from Eurisotop. All chemicals were used without prior purification.

Synthesis procedure of O,O'-(cyclohexane-1,4-diylbis(methylene)) dimethyl dioxalate

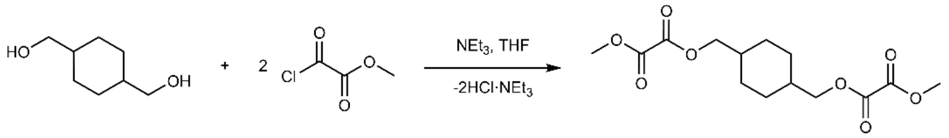

Methyl oxalyl chloride (3.675 g, 30.0 mmol, 2.0 equiv.) was dissolved in tetrahydrofuran (THF). To this, a solution of 1,4-cyclohexanedimethanol (2.163 g, 15.0 mmol, 1.0 equiv.) and triethylamine (3.066 g, 30.3 mmol, 2.02 equiv.), dissolved in approximately 30 ml THF, was slowly added with a dropping funnel over the course of one hour. During the addition, a white precipitate formed. Additional THF was added if the stirring of the reaction mixture was inhibited by the precipitate. After complete addition, the reaction mixture was stirred for another hour. The white precipitate was

filtered off and the THF was evaporated. The residual, viscous yellow liquid was redissolved in dichloromethane (DCM) and extracted with water and brine. The organic layer was dried over Na_2SO_4 and DCM was evaporated. Due to some remaining impurities detected by ^{1}H NMR, the white solid was washed with cyclohexane and diethyl ether, which predominately dissolved the *cis*-stereoisomer of the reaction product. In order to obtain a representative mixture of both *cis* and *trans* isomers of the reaction product, the cyclohexane-diethyl ether washing fraction was partially evaporated until approximately 10 ml of solvent remained. Some crystals were precipitating from the solution, and the flask was placed in the refrigerator overnight. The crystals were then filtered off and combined with the previously obtained crystals of the *trans* product. To ensure a homogenous reaction product, the crystals were dissolved in a small amount of DCM. After evaporation of the solvent, the white crystals (2.67 g, 56.3%) were dried *in vacuo*. A *cis*/*trans* ratio of 21/79 was determined by ^{1}H NMR.

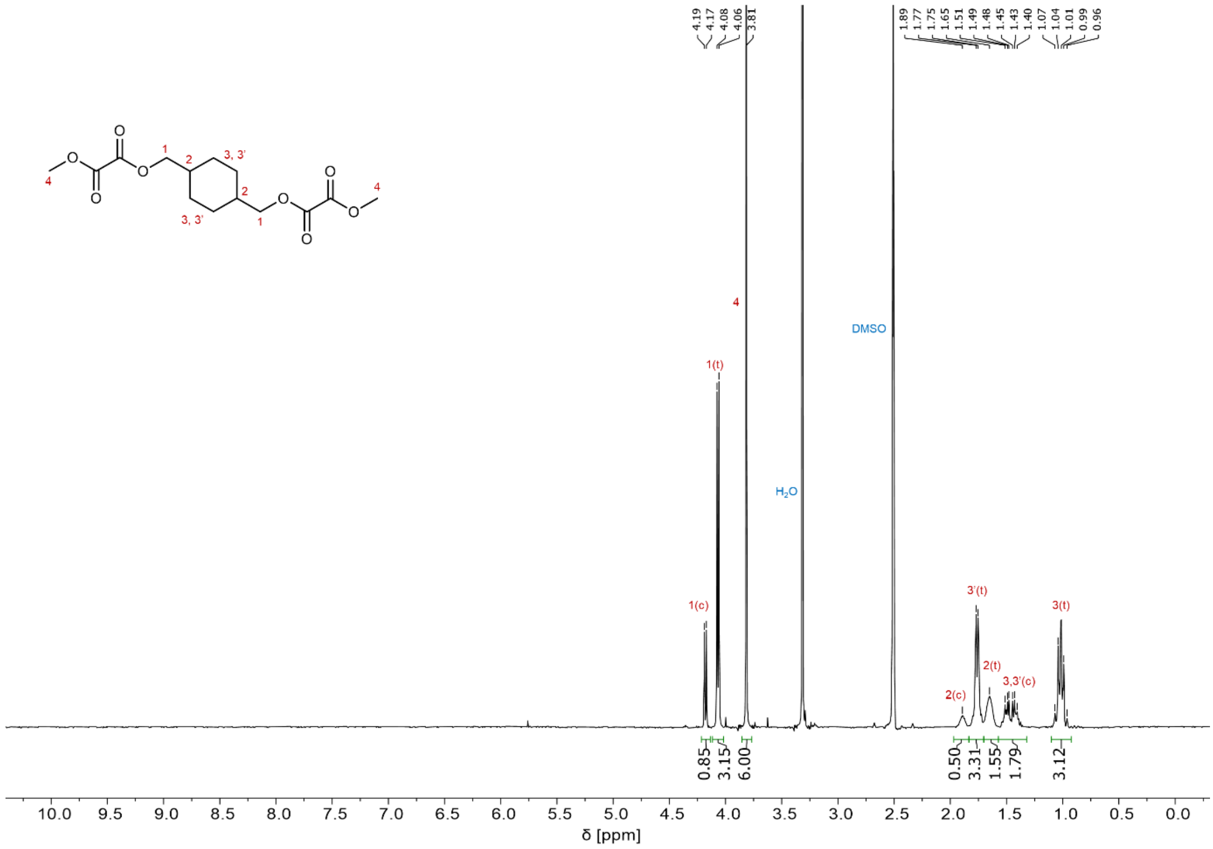

Figure S. 1 ^{1}H NMR spectrum of O,O'-(cyclohexane-1,4-diylbis(methylene)) dimethyl dioxalate in DMSO-d6.

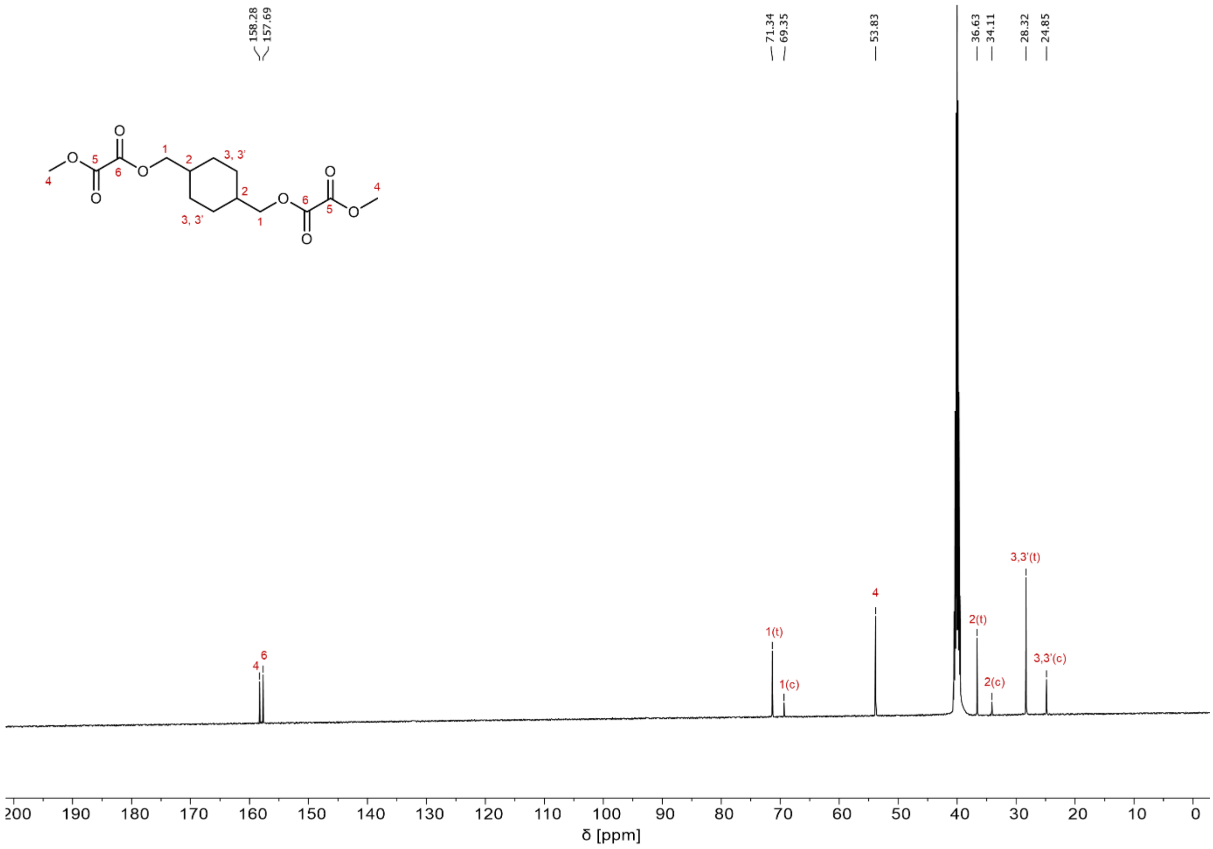

Figure S. 2 ^{13}C NMR spectrum of O,O'-(cyclohexane-1,4-diylbis(methylene)) dimethyl dioxalate in DMSO-d6.

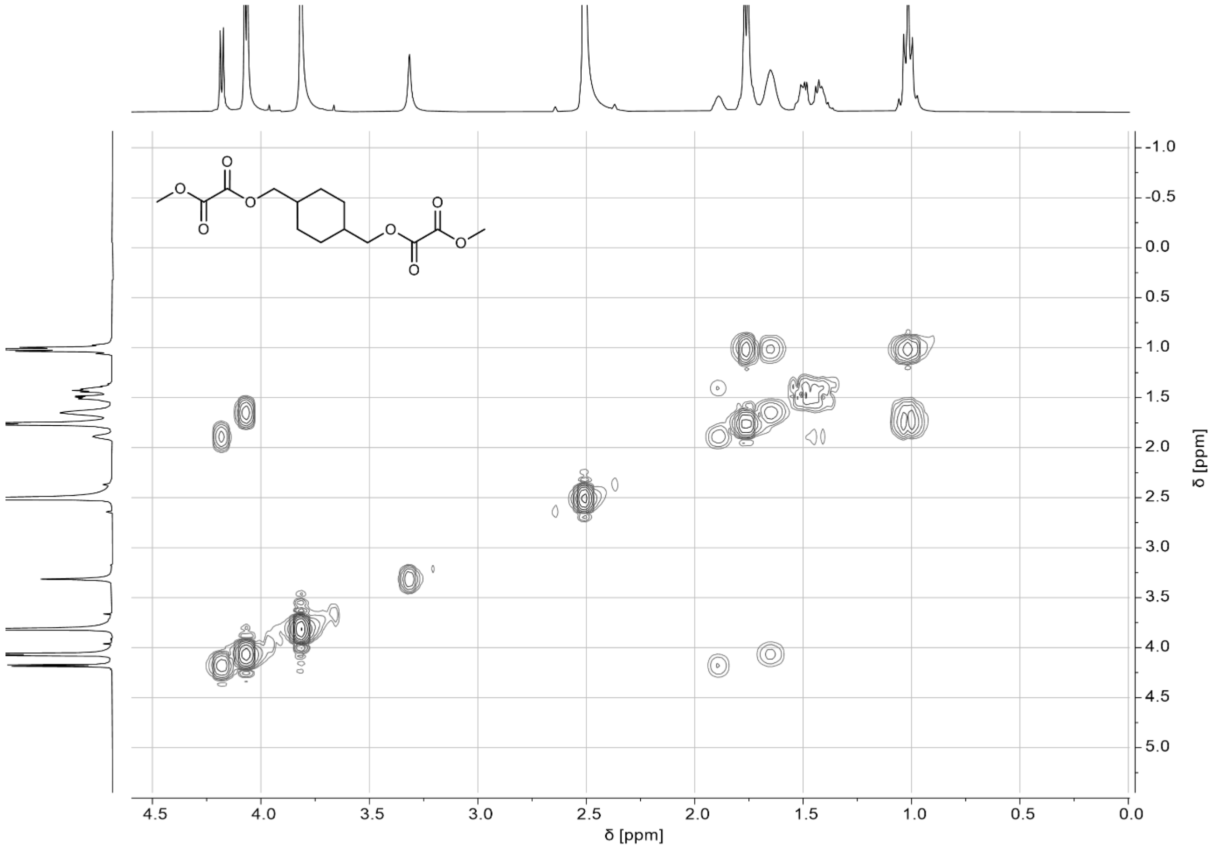

Figure S. 3 ^{1}H-^{1}H COSY NMR spectrum of O,O'-(cyclohexane-1,4-diylbis(methylene)) dimethyl dioxalate in DMSO-d6.

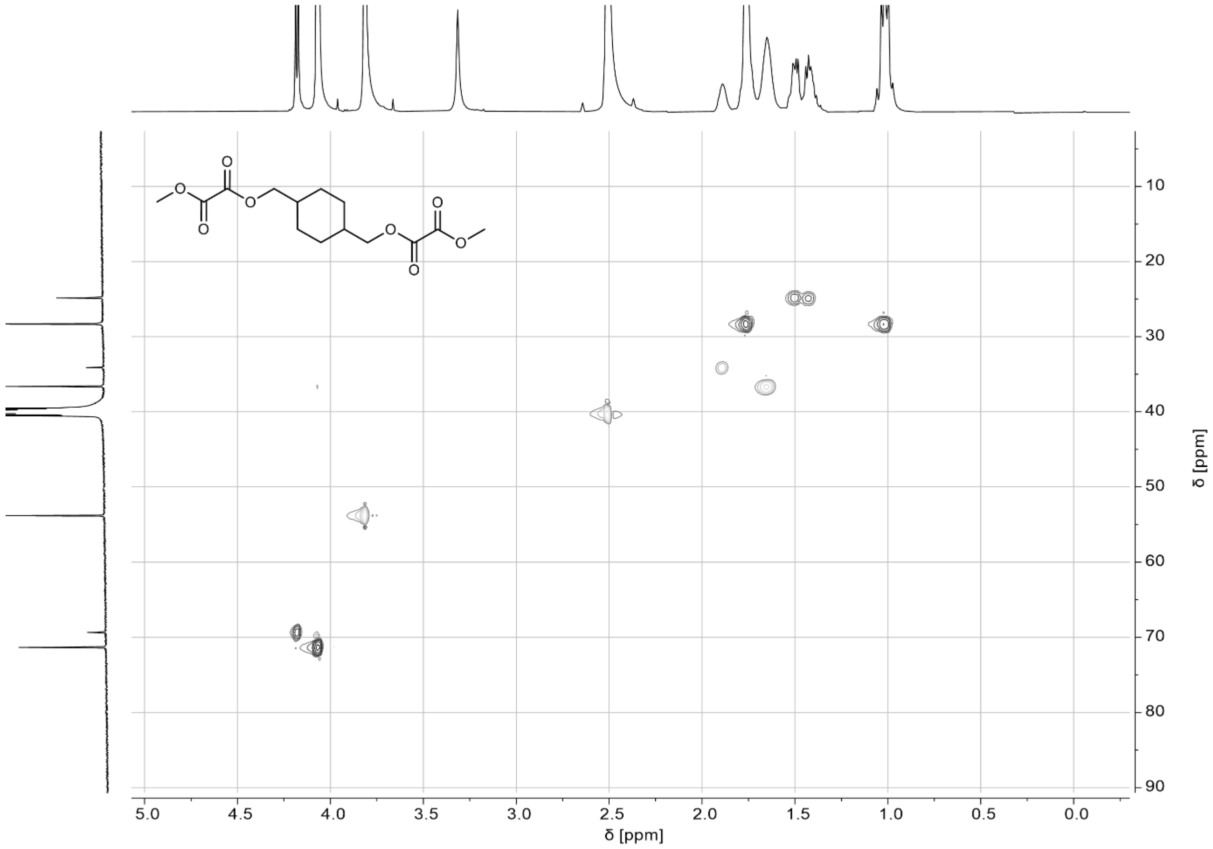

Figure S. 4 ^{1}H-^{13}C HSQC NMR spectrum of O,O'-(cyclohexane-1,4-diylbis(methylene)) dimethyl dioxalate in DMSO-d6.

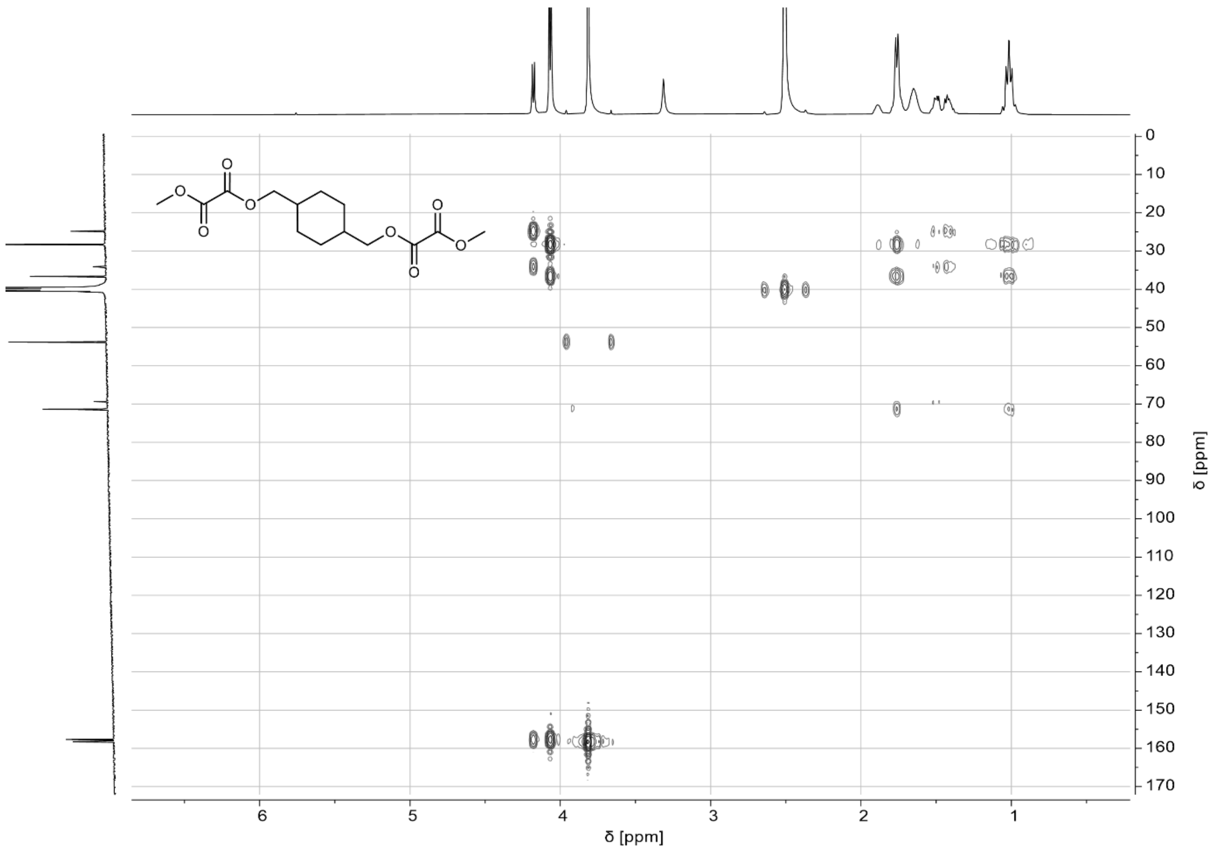

Figure S. 5 ^{1}H-^{13}C HMBC NMR spectrum of O,O'-(cyclohexane-1,4-diylbis(methylene)) dimethyl dioxalate in DMSO-d6.

Table S 1. Properties of soil.

Properties	Values
Soil type	Sandy loam
Particle size distribution (%): <0.002/0.002-0.05/0.05-2.0 mm	11/15/74
Organic carbon (mg g^{-1}, dry soil)	177±56
Nitrogen (mg g^{-1}, dry soil)	20±6
C:N (g C g^{-1} N)	9
Phosphate (µg g^{-1}, dry soil)	4.6
Cation exchange capacity (meq 100 g^{-1} dry soil)	8.5±2.0
Maximum water holding capacity (g water 100 g^{-1} dry soil)	43.3±5.1
pH (0.01 M $CaCl_2$)	4.9*

* Measured value. All other values were obtained from the supplier.

Table S 2. Constituents of mineral salts solution used to adjust soil moisture (OECD, 2014).

**Salts**	**mg L^{-1}**
Potassium dihydrogen phosphate (KH_2PO_4)	85.0
Dipotassium hydrogen phosphate (K_2HPO_4)	217.5
Disodium hydrogen phosphate dihydrate ($Na_2HPO_4.2H_2O$)	334.0
Ammonium chloride (NH_4Cl)	50.0
Calcium chloride dihydrate ($CaCl_2.2H_2O$)	36.40
Magnesium sulphate heptahydrate ($MgSO_4.7H_2O$)	22.50
Iron (III) chloride hexahydrate ($FeCl_3.6H_2O$)	0.25

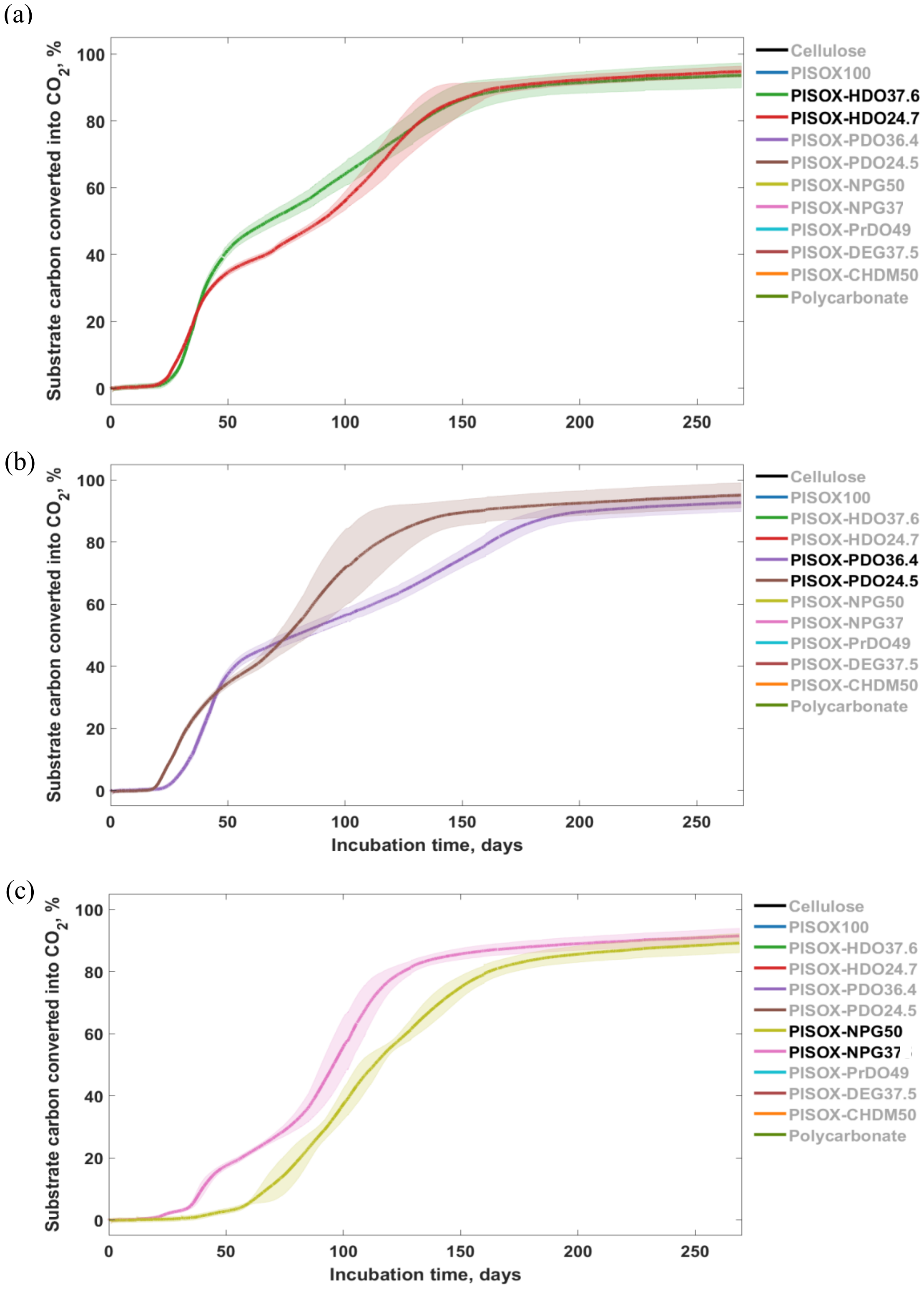

Figure S. 6 Biodegradation curves for PISOX copolyesters with 1,6-hexanediol (HDO) (a), 1,5-pentanediol (PDO) (b) and neopentyl glycol (NPG) (c) as co-diol. Mean biodegradation (lines) were plotted and the shaded area represents the standard deviation of at least four replicates for each polymer composition.

(a)

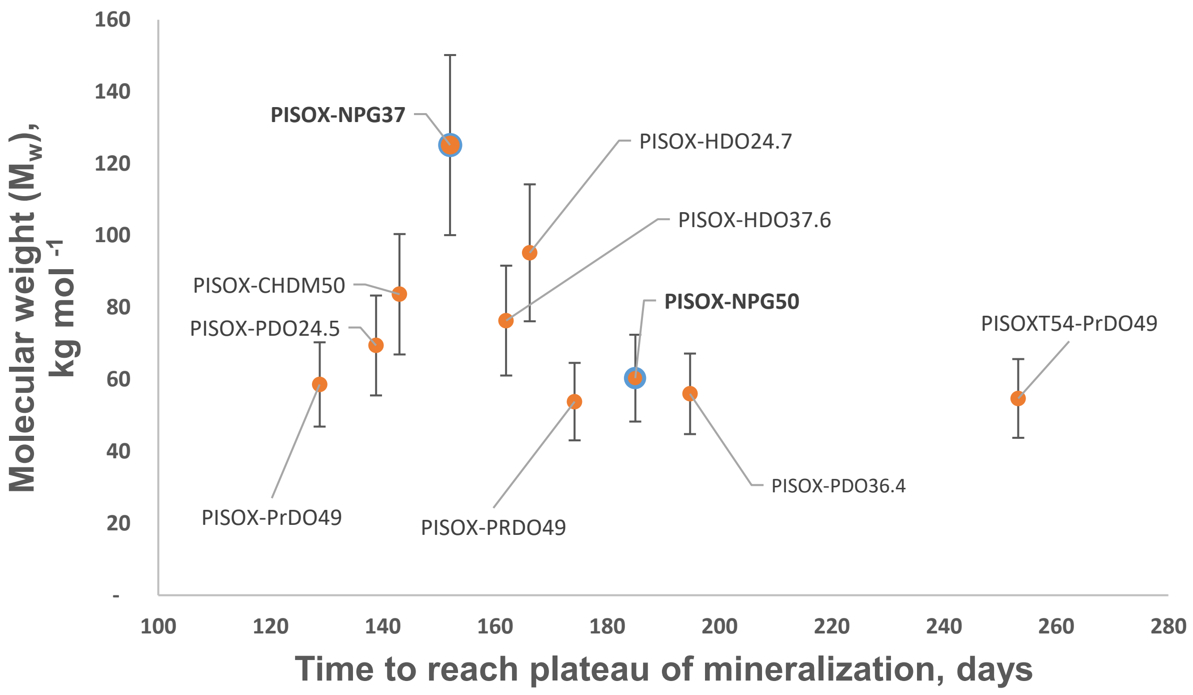

Molecular weight (M_w), kg mol -1
Time to reach plateau of mineralization, days
PISOX-NPG37
PISOX-HDO24.7
PISOX-HDO37.6
PISOX-CHDM50
PISOX-PDO24.5
PISOX-NPG50
PISOXT54-PrDO49
PISOX-PrDO49
PISOX-PRDO49
PISOX-PDO36.4
160
140
120
100
80
60
40
20
-
100
120
140
160
180
200
220
240
260
280

(b)

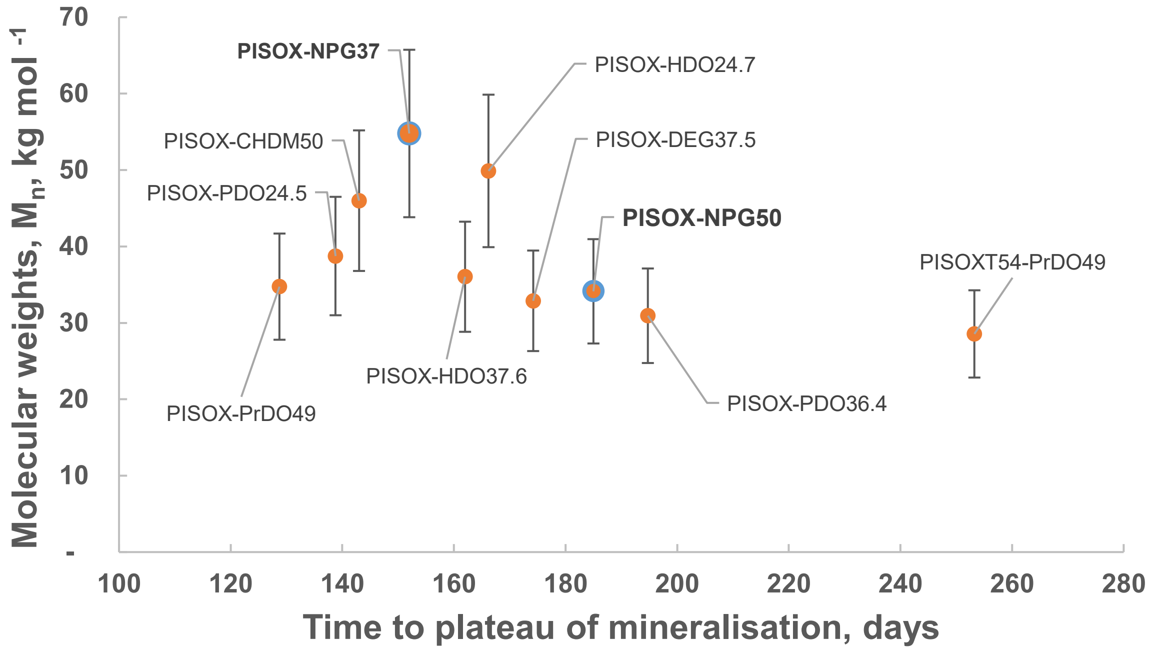

Molecular weights, M_n, kg mol -1
Time to plateau of mineralisation, days
PISOX-NPG37
PISOX-HDO24.7
PISOX-CHDM50
PISOX-DEG37.5
PISOX-PDO24.5
PISOX-NPG50
PISOXT54-PrDO49
PISOX-HDO37.6
PISOX-PrDO49
PISOX-PDO36.4
70
60
50
40
30
20
10
-
100
120
140
160
180
200
220
240
260
280

(c)

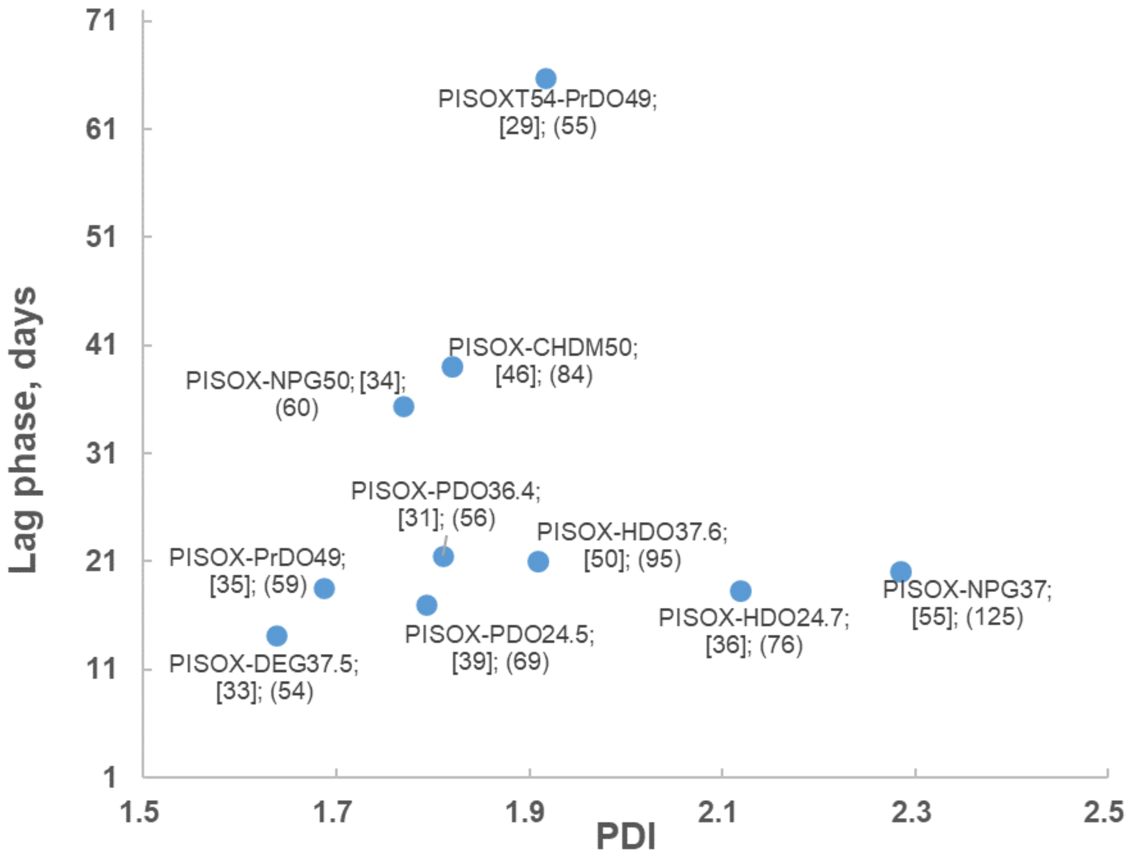

Figure S. 7 The molecular weights (M_n,(a); M_w, (b)) v.s. the time to plateau of mineralisation (daily mineralisation rate < 0.1 mg) (average of replicates) for PISOX copolyesters. Error bars represent 20% difference respectively. PISOX is not included in the figure as it is insoluble in DCM; it plateaus at 130 days. (c) The polydispersity index (PDI) v.s. the lag phase of mineralisation (average of replicates) for PISOX copolyesters. Corresponding Mn and Mw values (in kg mol $^{-1}$) are labeled in square brackets [] and parentheses () respectively.

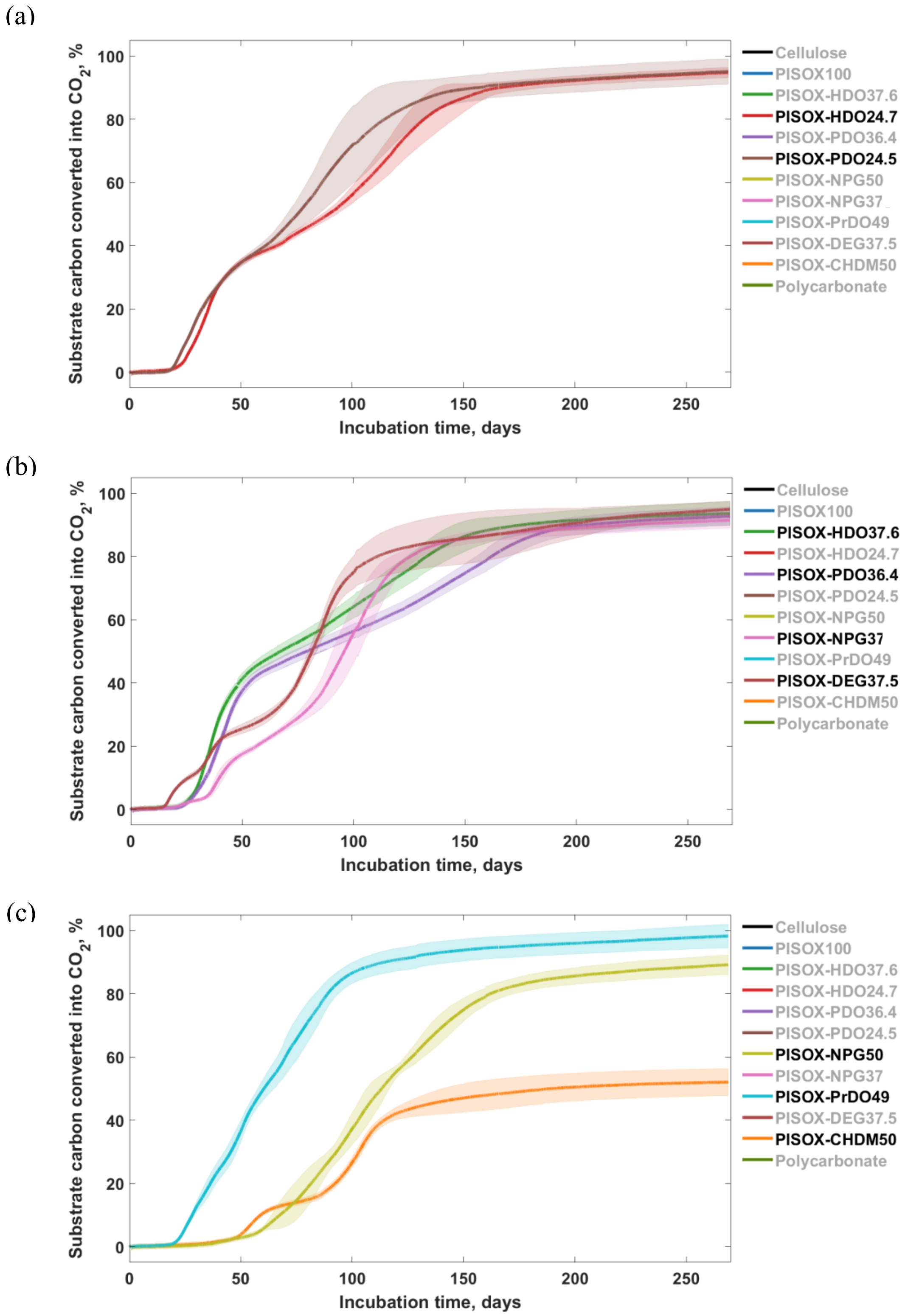

Figure S. 8 Biodegradation curves of PISOX copolyesters with the similar co-diol ratio (25/75 (a), 37.5/62.5 (b), and 50/50 (c)) at 25 °C in soil. Mean biodegradation (lines) were plotted. The shaded area represents the standard deviation of at least three replicates.

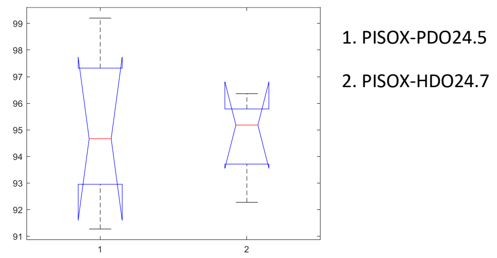

(a) PISOX-diol25, **p>0.05**

Source	SS	df	MS	F	Prob>F
Groups	0.2287	1	0.22869	0.03	0.857
Error	45.7719	7	6.53884		
Total	46.0006	8			

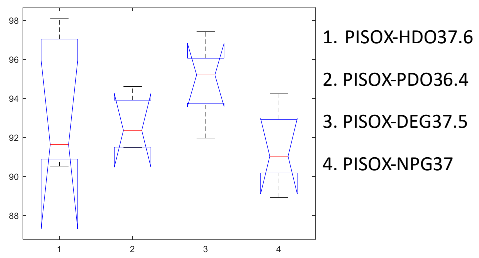

(b) PISOX-diol37.5, **p>0.05**

Source	SS	df	MS	F	Prob>F
Groups	31.537	3	10.5123	1.78	0.1938
Error	88.517	15	5.9011		
Total	120.054	18			

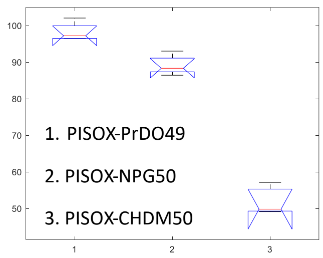

(c) PISOX-diol50, **p<0.05**

Source	SS	df	MS	F	Prob>F
Groups	3997.28	2	1998.64	206.79	3.00267e-08
Error	86.99	9	9.67		
Total	4084.27	11			

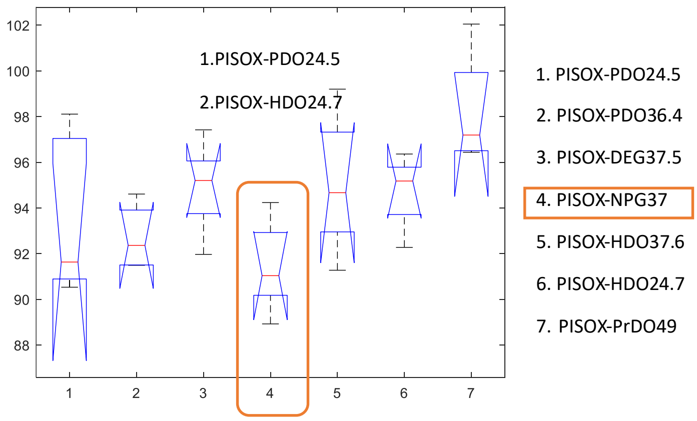

(d) PISOX-diol25&37.5 and PrDO49, **p<0.05**

Source	SS	df	MS	F	Prob>F
Groups	120.065	6	20.0109	3.23	0.0173
Error	154.994	25	6.1997		
Total	275.059	31			

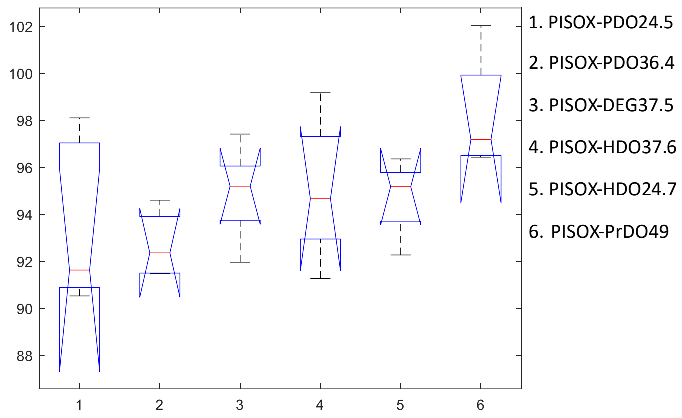

(e) All linear co-diol (PISOX-NPG excluded), **p>0.05**

Source	SS	df	MS	F	Prob>F
Groups	71.912	5	14.3824	2.17	0.0959
Error	138.883	21	6.6135		
Total	210.794	26			

Figure S. 9 Boxplots of the biodegradation percentages for PISOX copolyesters with 25% (a), 37.5% (b), 50% (c), all 25% & all 37.5% & PrDO 49% (d), all linear (e) co-diol, respectively. Highlighted group in (d) was excluded in (e). The central red mark, the bottom and top edges of the box indicate the median, and the 25th and 75th percentiles, respectively. The lines extending from the box represent the variability outside the upper and lower quartiles. Corresponding ANOVA table shown under the graphs.

	Before incubation	After incubation
PISOX-CHDM50 (a)	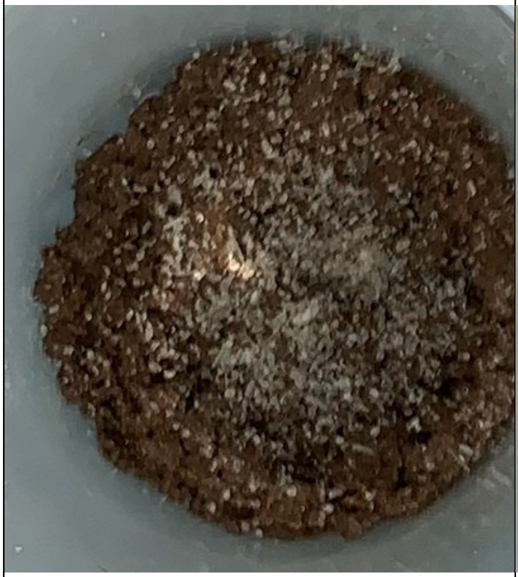	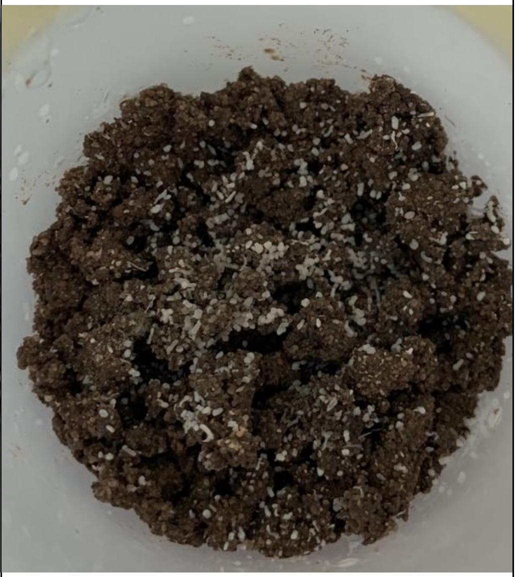
PISOXT54-PrDO49 (b)	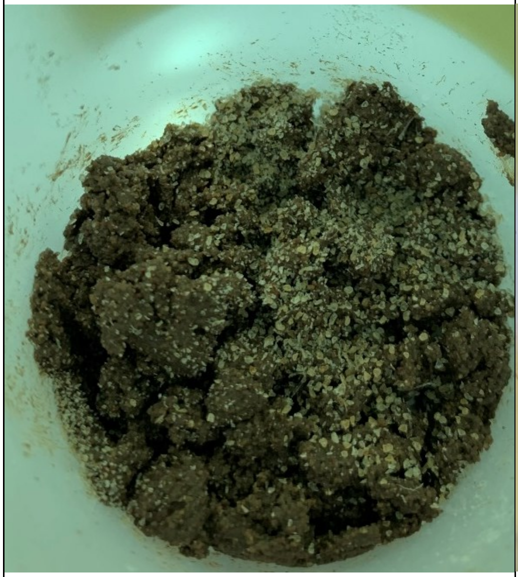	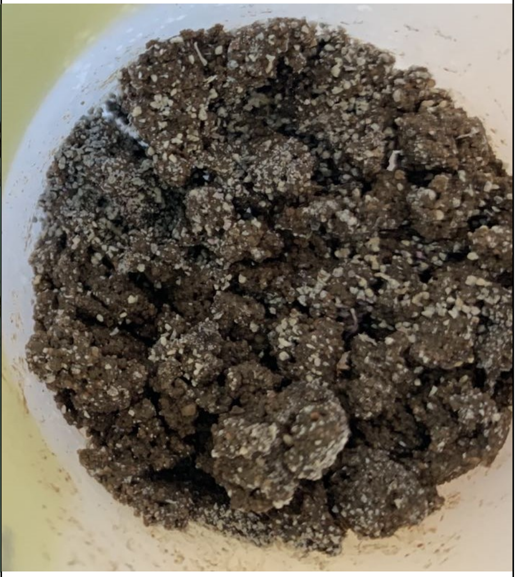

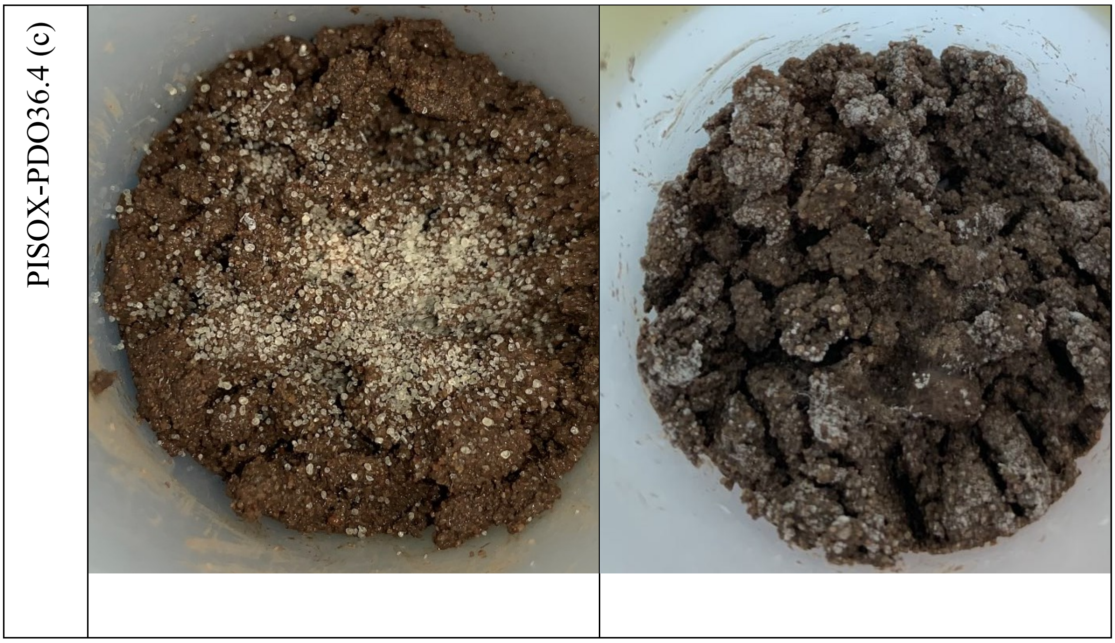

Figure S. 10 Photos of polymers before and after incubation. Visible particles left for PISOX-CHDM50 (a) and PISOXT54-PrDO49 (b) after incubation. Disappearance of particles and growth of microorganisms was observed for PSIXO-PDO36.4 (c).

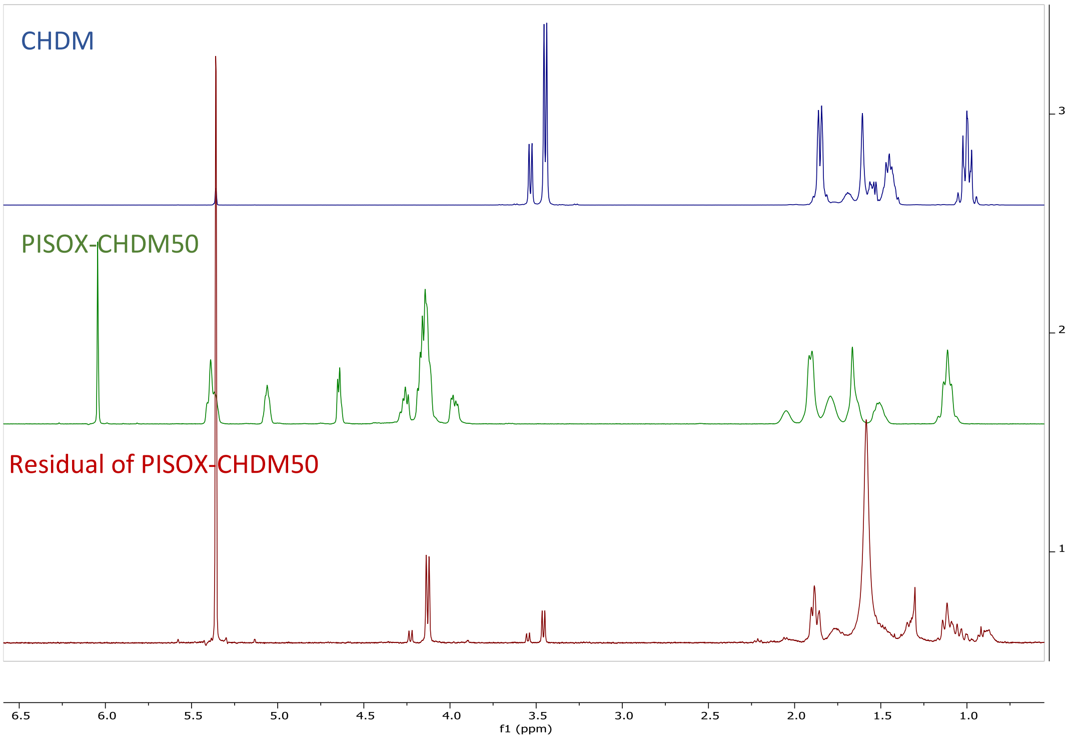

Figure S. 11 ^{1}H NMR spectra of 1,4-cyclohexanedimethanol (in DCM-d2), PISOX-CHDM50 (in TCE, from[1]) and the residual of PISOX-CHDM50 after biodegradation (in DCM-d2). The *cis/trans* ratios of CHDM-monomer (3.44-3.56) and CHDM-esters (4.10-4.25 ppm) are 21/79 and 9/91 respectively.

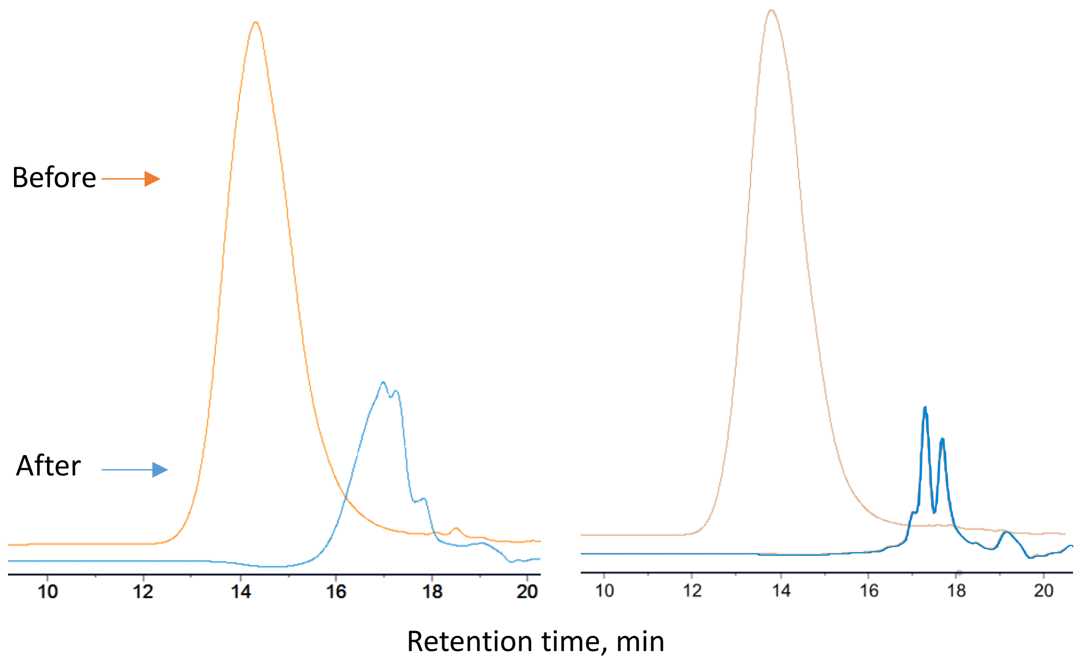

Figure S. 12 GPC chromatograms of PISOX-CHDM50 and PISOXT54-PrDO49 before and after biodegradation.

The molecular weight and molecular weight distribution of all polymers was measured by gel permeation chromatography (GPC, size exclusion chromatography on an Agilent HPLC system (1260 Infinity II)). It is equipped with two PL gel 5 µm MIXED-C (300 x 7.5 mm) columns and a refractive index detector. Dichloromethane (DCM) was used as mobile phase (1 mL min^{-1} at 35 °C) and polystyrene standards (Sigma Aldrich) were used for calibration. PISOX100 does not dissolve in DCM, therefore no GPC data available.

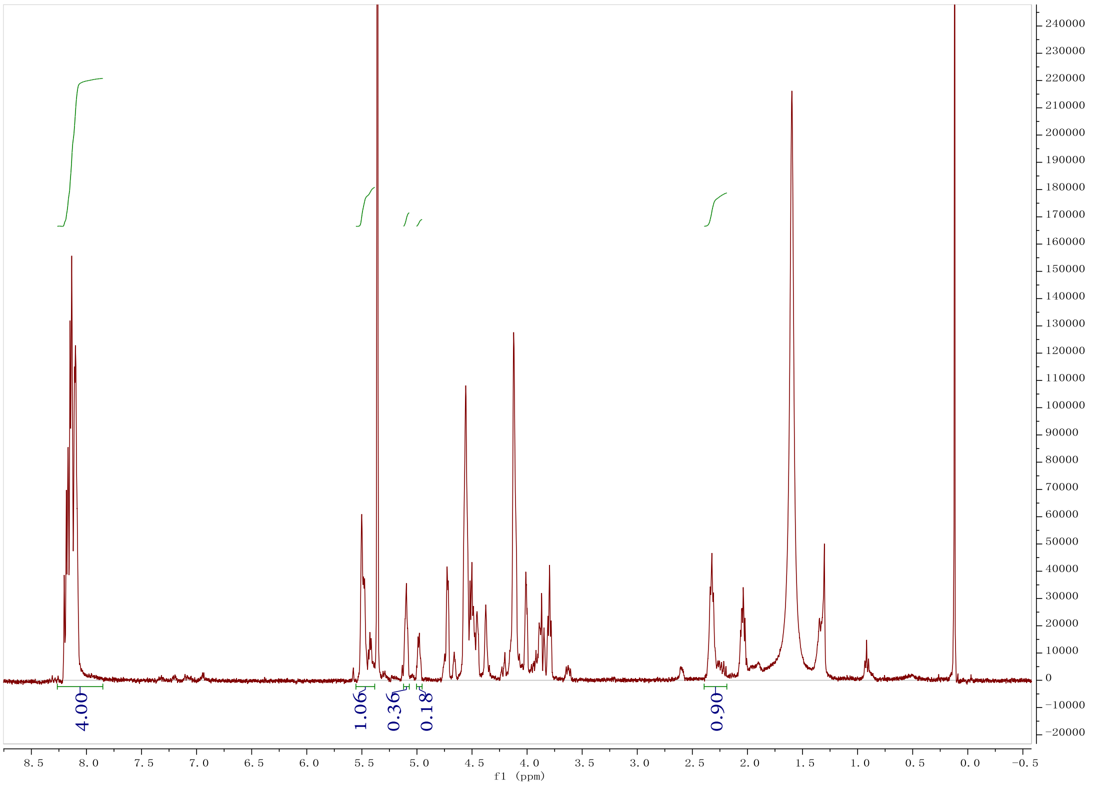

Figure S. 13 ^{1}H NMR spectrum of PISOXT54-PrDO49 after biodegradation in DCM-d2.

The peaks of 8.0-8.2, 5.4-5.6, 2.3-2.4 ppm represent TPA (4H), IS (2H) and PrDO (2H) units respectively. The sum integration of IS and PrDO (1.06/2+0.90/2=0.98) peaks is similar to that of TPA (4.00/4=1.00) (ratio,98:100), which suggests that the amount of OX units decreased significantly after biodegradation comparing to their original ratio before biodegradation (54:100).

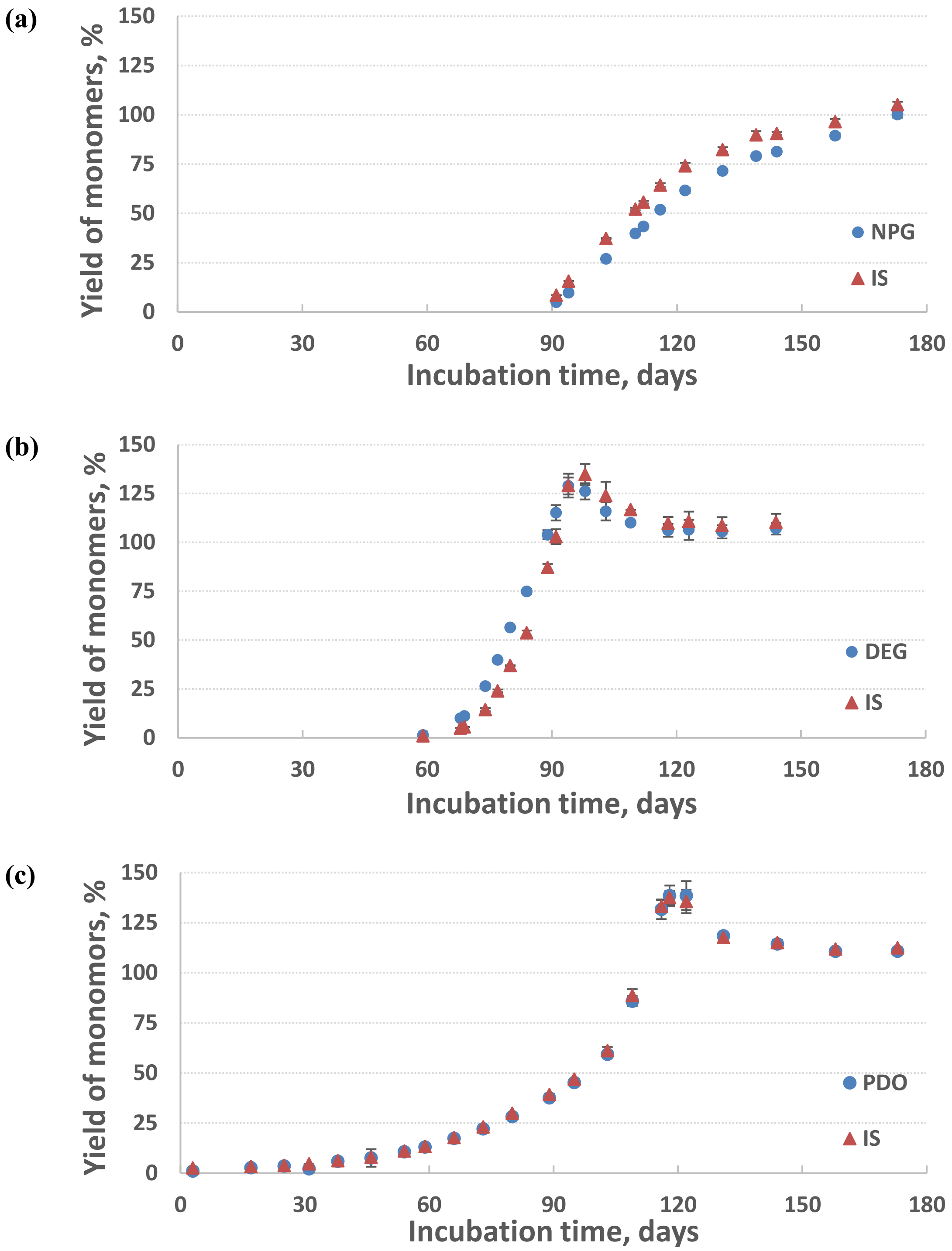

Figure S. 14 Individual yield of monomers, isosorbide and co-diol for PISOX-DEG37.5 (a), PISOX-NPG37 (b) and PISOX-PDO36.4 (c) during 6-month hydrolysis at 25 °C in D_2O as percentage of their theoretical maximum release. Error bars represent standard deviation of triplicate hydrolysis experiments. * Percentages of dissolved NPG and DEG relative to the total amount of hydrolysed diols in time are shown in Figure S. 16.

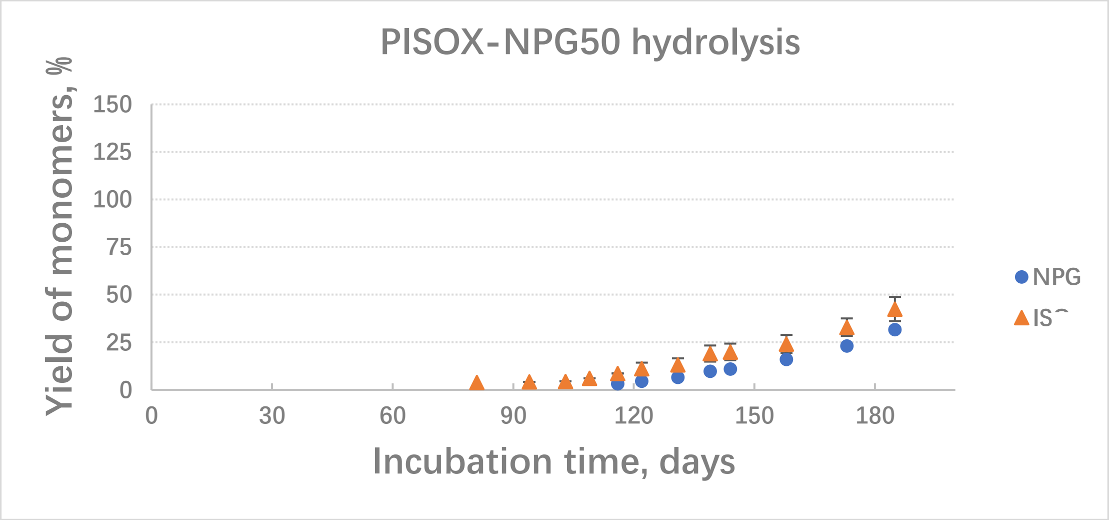
PISOX-NPG50 hydrolysis
Yield of monomers, %
150
125
100
75
50
25
0
0
30
60
90
120
150
180
Incubation time, days
NPG
IS

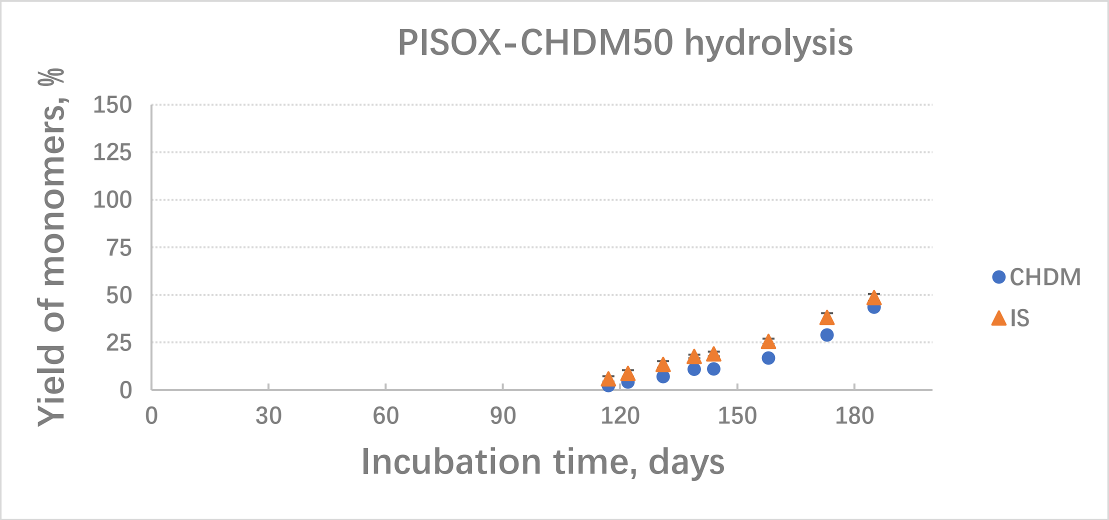
PISOX-CHDM50 hydrolysis
Yield of monomers, %
150
125
100
75
50
25
0
0
30
60
90
120
150
180
Incubation time, days
CHDM
IS

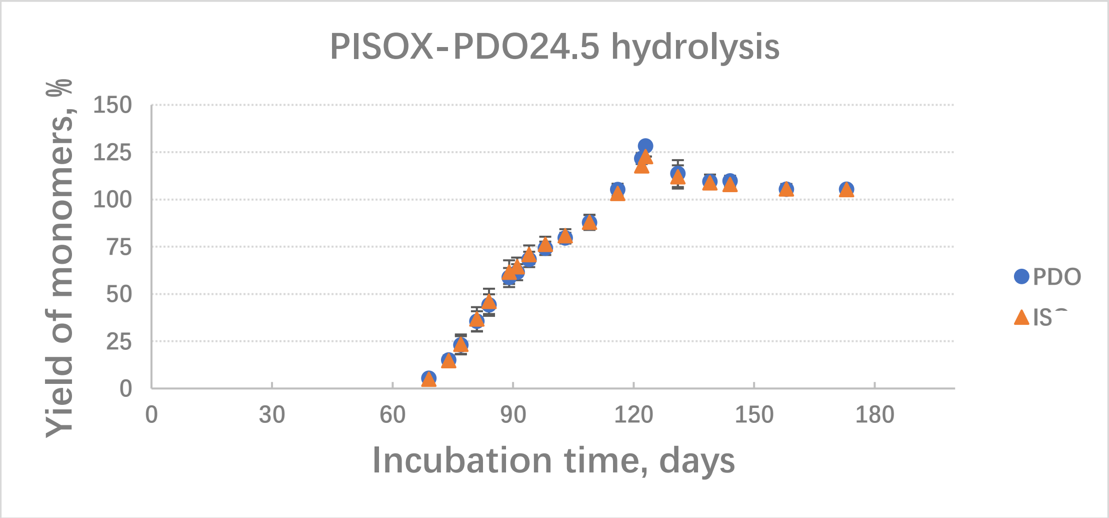
PISOX-PDO24.5 hydrolysis
Yield of monomers, %
150
125
100
75
50
25
0
0
30
60
90
120
150
180
Incubation time, days
PDO
IS

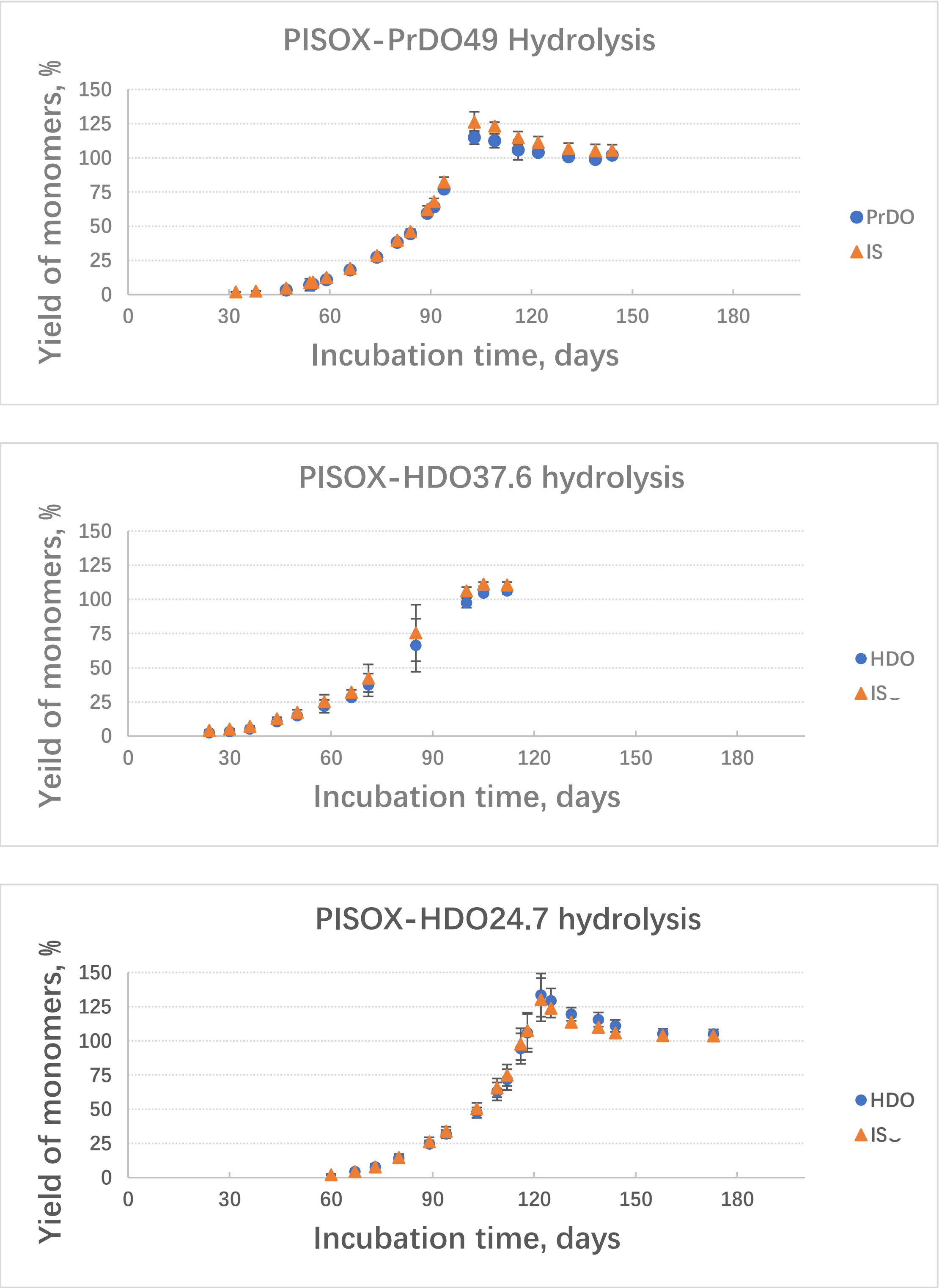

Figure S. 15 Individual yield of monomers, isosorbide and co-diol for PISOX copolyesters during 6-month hydrolysis at 25 °C in D_2O as percentage of their theoretical maximum release. Error bars represent standard deviation of triplicate hydrolysis experiments.

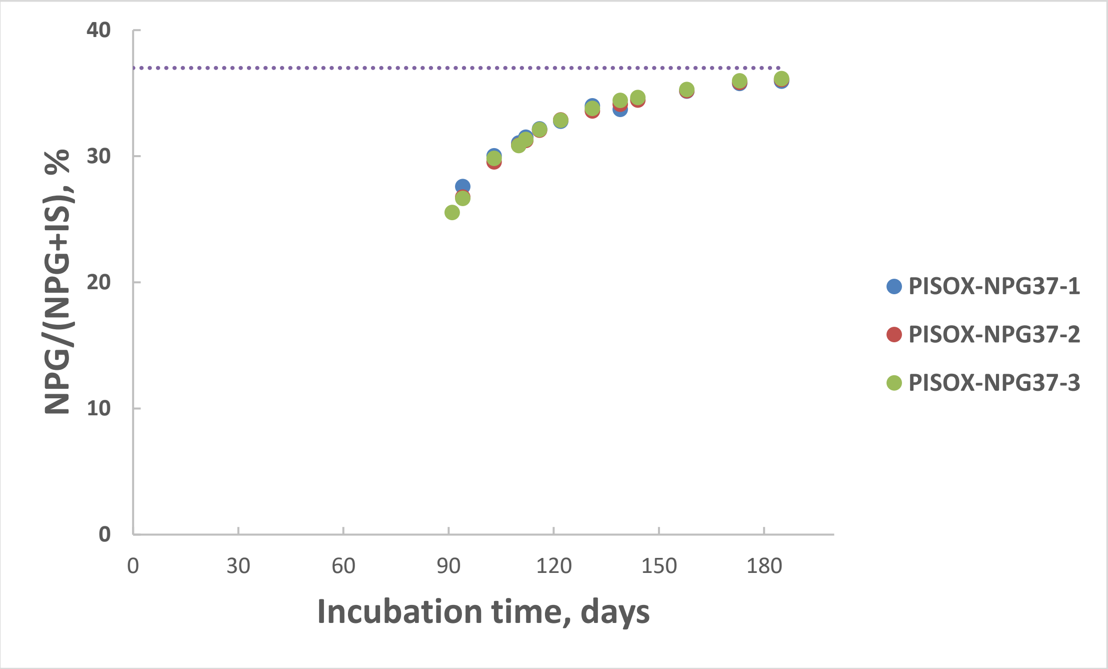

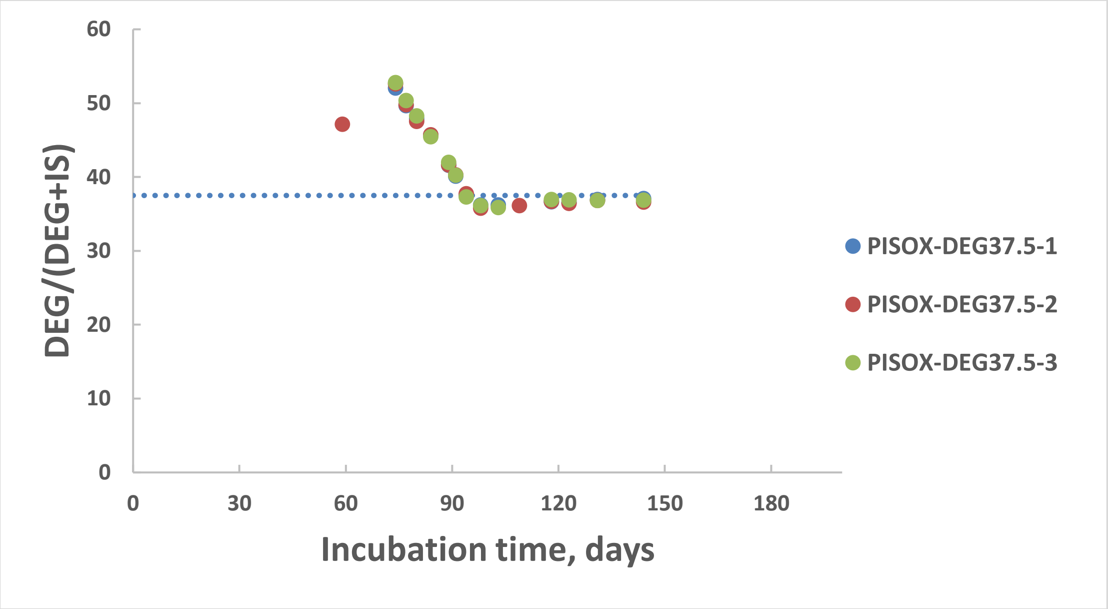

Figure S. 16 Percentages of dissolved NPG and DEG relative to the total amount of hydrolysed diols in time. Triplicates were plotted. Dotted lines show the composition in the polymer.

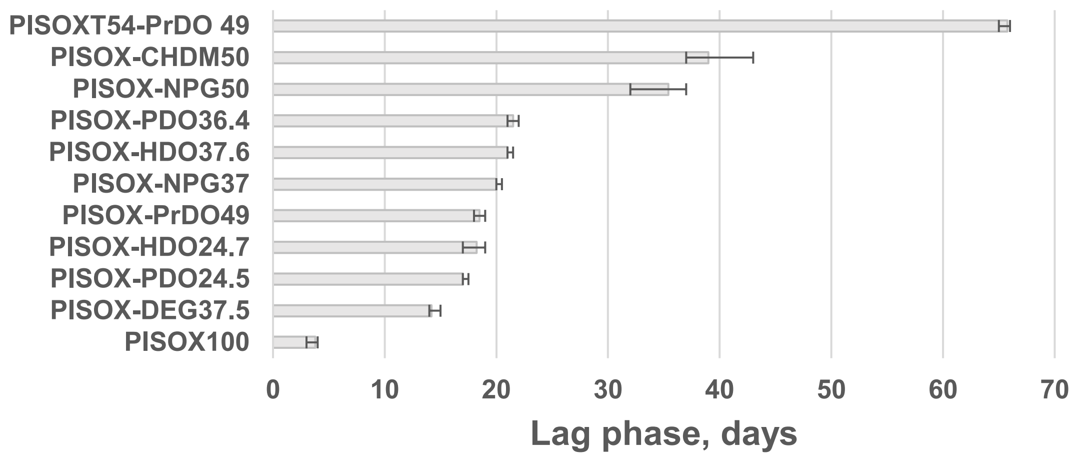

Figure S. 17 Overview of the lag phase for PISOX-based (co)polyesters biodegradation curves. Error bars represent variation of replicates.

The shortest lag phase of PISOX100 homopolyester and the fact that PISOX-NPG37 has a shorter lag phase than PISOX-NPG50 suggests that a higher isosorbide content versus neopentyl glycol (NPG) favours biodegradation due to the strong hydrophilicity/hygroscopicity of isosorbide. The small variations in lag phase of PISOX-PrDO, PISOX-PDO, PISOX-HDP show that intermediate chain length of the co-diol (i.e. C3 to C6, and the ratio from 25% to 50%) have little effect on the biodegradability of PISOX copolyesters. The relatively short lag phase of PISOX-DEG37.5 suggests that the presence of oxygen in ether diols could affect the interaction between polymer and enzyme to facilitate biodegradation. The relatively long lag phase of PISOX-NPG50 (especially compared to PISOX-PrDO50) and the slow non-enzymatic hydrolysis may be attributed to the higher steric hindrance (relative to the linear structure) as a result of the two methyl branches.

The cyclic building blocks are too apolar (hydrophobic) and may thus hinder the biodegradation/hydrolysis of the copolyesters. As a result, the lag phase of PISOX-CHDM50 and PISOXT54-PrDO49 increased. Compared to PISOX-PrDO49, the lag phase of the biodegradation curve for PISOXT54-PrDO49 was notably prolonged (over three times to 70 days). This resulted from replacing easily hydrolysable ester bonds (i.e. oxalic esters) with aromatic acid esters (i.e. terephthalate esters). This supports the conclusion that oxalate esters can play a crucial role in faster biodegradation.

Reference

(1) van der Maas, K. Aryl-Oxalates - a Versatile and Highly Reactive Monomer for Polyester Synthesis (in Preparation), University of Amstedam, 2024.
